# Nanosecond heme-to-heme electron transfer rates in a multiheme cytochrome nanowire reported by a spectrally unique His/Met-ligated heme

**DOI:** 10.1073/pnas.2107939118

**Published:** 2021-09-23

**Authors:** Jessica H. van Wonderen, Katrin Adamczyk, Xiaojing Wu, Xiuyun Jiang, Samuel E. H. Piper, Christopher R. Hall, Marcus J. Edwards, Thomas A. Clarke, Huijie Zhang, Lars J. C. Jeuken, Igor V. Sazanovich, Michael Towrie, Jochen Blumberger, Stephen R. Meech, Julea N. Butt

**Affiliations:** ^a^School of Chemistry, University of East Anglia, Norwich Research Park, Norwich NR4 7TJ, United Kingdom;; ^b^Department of Physics and Astronomy, University College London, London WC1E 6BT, United Kingdom;; ^c^Thomas-Young Centre, University College London, London WC1E 6BT, United Kingdom;; ^d^School of Biological Sciences, University of East Anglia, Norwich Research Park, Norwich NR4 7TJ, United Kingdom;; ^e^School of Biomedical Sciences, Faculty of Biological Sciences, University of Leeds, Leeds LS2 9JT, United Kingdom;; ^f^Central Laser Facility, Research Complex at Harwell, Rutherford Appleton Laboratories, Science and Technology Facilities Council, Harwell Science Campus, Didcot OX11 0QX, United Kingdom

**Keywords:** electron transfer, Moser–Dutton ruler, pump-probe spectroscopy, Ru(II)tris(bipyridine), *Shewanella*

## Abstract

Proteins achieve efficient energy storage and conversion through electron transfer along a series of redox cofactors. Multiheme cytochromes are notable examples. These proteins transfer electrons over distance scales of several nanometers to >10 μm and in so doing they couple cellular metabolism with extracellular redox partners including electrodes. Here, we report pump-probe spectroscopy that provides a direct measure of the intrinsic rates of heme–heme electron transfer in this fascinating class of proteins. Our study took advantage of a spectrally unique His/Met-ligated heme introduced at a defined site within the decaheme extracellular MtrC protein of *Shewanella oneidensis*. We observed rates of heme-to-heme electron transfer on the order of 10^9^ s^−1^ (3.7 to 4.3 Å edge-to-edge distance), in good agreement with predictions based on density functional and molecular dynamics calculations. These rates are among the highest reported for ground-state electron transfer in biology. Yet, some fall 2 to 3 orders of magnitude below the Moser–Dutton ruler because electron transfer at these short distances is through space and therefore associated with a higher tunneling barrier than the through-protein tunneling scenario that is usual at longer distances. Moreover, we show that the His/Met-ligated heme creates an electron sink that stabilizes the charge separated state on the 100-μs time scale. This feature could be exploited in future designs of multiheme cytochromes as components of versatile photosynthetic biohybrid assemblies.

## Significance

Multiheme cytochromes have been identified as essential proteins for electron exchange between bacterial enzymes and redox substrates outside of the cell. In microbiology, these proteins contribute to efficient energy storage and conversion. For biotechnology, multiheme cytochromes contribute to the production of green fuels and electricity. Furthermore, these proteins inspire the design of molecular-scale electronic devices. Here, we report exceptionally high rates of heme-to-heme electron transfer in a multiheme cytochrome. We expect similarly high rates, among the highest reported for ground-state electron transfer in biology, in other multiheme cytochromes as the close-packed hemes adopt similar configurations despite very different amino acid sequences and protein folds.

Electron transfer (ET) along a series of protein cofactors over several nanometers is widely used for energy storage and conversion in biology (1, 2). Prominent examples are found in respiratory complexes of the inner mitochondrial membrane, which couple exergonic electron flow to endergonic transmembrane proton translocation, and in light-harvesting Photosystems I and II, that enable rapid separation of the photochemically created electron–hole pair. Typically, the redox-active protein cofactors are separated by ≥1 nm (edge-to-edge), and the space between them is occupied by components of the protein matrix (3, 4). A different situation is found in multiheme cytochromes (5, 6). For these proteins, a defining feature is close-packed *c*-type hemes often in van der Waals contact and arranged as approximately linear chains that span the tertiary structure.

Multiheme cytochromes are versatile ET modules, and interactions between two or more proteins can extend the distances accessible to ET. Notable examples are found in electromicrobiology where intracellular ET is coupled to transformation of extracellular redox partners. In the MTR complex of *Shewanella* species, two multiheme cytochromes, MtrA and MtrC, are positioned to move electrons >16 nm across the cell envelope (7). Extracellular electrically conductive nanowires of *Geobacter* with lengths exceeding 10 μm are comprised solely of the repeated hexaheme-containing OmcS (8, 9). Respiratory ET through these proteins harnesses energy from chemically stratified environments and contributes to the global cycling of Fe, Mn, N, and S (10). Beyond these contributions to microbiology, such ET contributes to the bacterial remediation of contaminated soils and to microbial strategies for the production of clean electricity, fuels, and fine chemicals (11–13). When purified, the multiheme cytochromes also attract much attention for their potential utilization in bioelectronics (6, 14–18).

A survey of multiheme cytochromes isolated from many species and cellular locations reveals the majority of hemes have His/His axial ligation and that nearest neighbors typically adopt one of two heme-packing motifs (5, 7–9, 19). In the T-shaped motif, porphyrin rings of neighboring sites are approximately orthogonal with edge-to-edge distances of ∼0.6 nm. In the parallel stacked packing motif, the neighboring porphyrin rings lie parallel and in van der Waals contact. The proximities of these centers suggest that ET between neighboring sites may lie at the higher end of those reported for intra- and interprotein ET. Unfortunately, however, the intrinsic ET rates across multiheme cytochromes and their complexes have not yet been determined experimentally with one exception (20), the small tetraheme cytochrome STC (see next paragraph). For the larger, biotechnologically relevant multiheme cytochromes including MtrC, MtrF, MtrA, and OmcS only computational estimates are available (21, 22). This situation represents a major gap in our empirical functional knowledge of these proteins; a molecular-scale understanding of the limiting factors for bacterial respiration and biotechnologies dependent on multiheme cytochromes is lacking. From a fundamental perspective, it is also of interest to consider how the ET rates in multiheme cytochromes compare to those predicted by the Moser–Dutton ruler (M-DR) where the *ΔG*-optimized ET rates show an exponential decay with tunneling distance that is largely independent of the nature of the redox-active centers and the intervening protein structure (3, 4). While the M-DR is known to give good estimates for long range biological tunneling (∼0.8 to 2 nm), it is not clear whether this simple relationship extends to multiheme cytochromes where tunneling distances are shorter (0.36 to 0.8 nm). Deviations from exponential distance dependence are typically 1 to 2 orders of magnitude (3, 4, 23, 24) and could in some cases be explained in terms of the tunneling pathway model (25, 26).

**Fig. 1.**
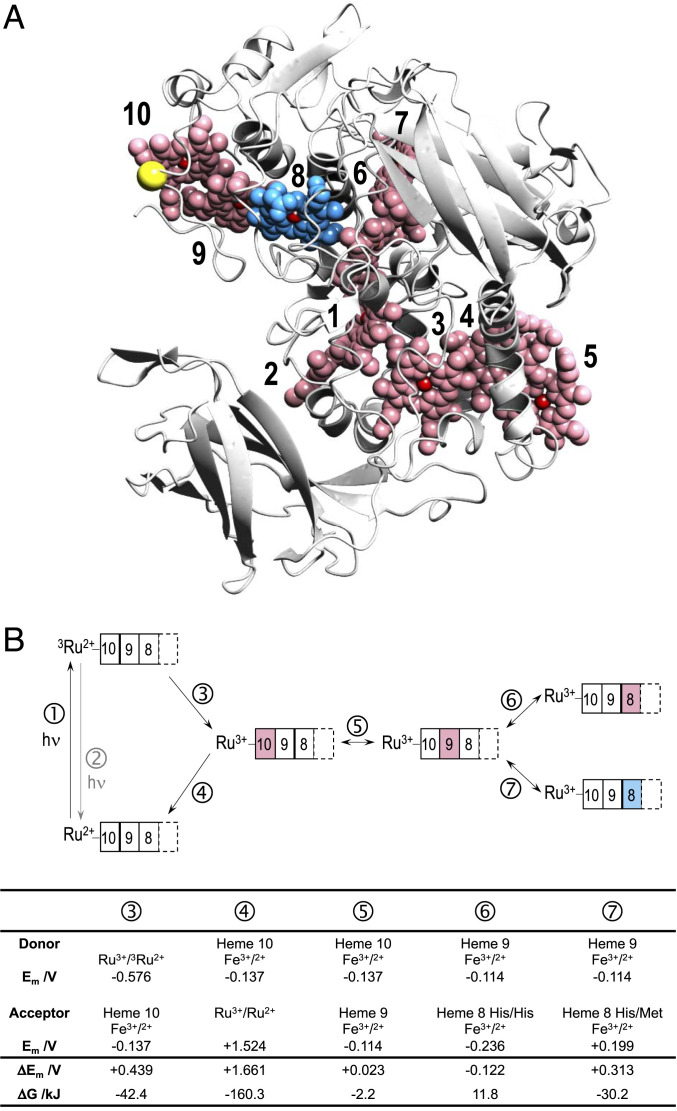
(*A*) Structure of MtrC. The 10 *c*-type hemes (red/blue spheres) are numbered in order of attachment from N to C terminus. Heme 8 (blue) has His/Met coordination in Ru-MtrC Met8. Residue 657 (yellow sphere) provides the site of Ru-dye attachment, see *Results* for details. (Protein Data Bank code: 4LM8). (*B*) Overview of photochemistry and heme–heme ET in the Ru-MtrC proteins of this work. Photon absorption ① and emission ②. Charge separation ③ and recombination ④. ET between hemes (rectangles) indicated for Fe(III) (open) and Fe(II) (filled) states for the Heme 10↔9 pair ⑤ and the Heme 9↔8 pair of Ru-MtrC His8 ⑥ and Ru-MtrC Met8 ⑦. His/His (red) and His/Met (blue) ligated hemes are indicated. Thermodynamic parameters relevant to the indicated processes are shown. *E*_m_ values are versus SHE: for the Ru-dye from ref. 30; for His/Met Heme 8 measured as described in this work; for the His/His ligated hemes are microscopic reduction potentials calculated as previously described (21).

In previous work, we demonstrated the feasibility of using pump-probe spectroscopy to investigate heme–heme ET in STC (20). Electron injection into the all-oxidized Fe(III)-heme chain occurred following photoexcitation of a Ru(II)(bipyridine)_3_-dye attached to the protein surface near a terminus of the heme chain. Fitting the resultant time trace of reduced Fe(II)-heme to a kinetic model, we were able to provide experimental estimates for ET rates in multiheme cytochromes. However, direct evidence for ET along the heme chain was lacking as we were unable to distinguish the reduced forms of the four His/His-ligated hemes in the tetraheme chain. Here, we overcome that limitation through studies of a multiheme cytochrome engineered to contain a spectroscopically unique His/Met-ligated heme at a defined site in the heme chain, Fig. 1. Our results provide unprecedented insight into the rates of heme-to-heme ET in multiheme cytochromes. The very high rates observed, on the order of 10^9^ s^−1^, are among the highest reported for ground-state ET in biology.

## Results

Pump-probe spectroscopy was performed on engineered forms of the decaheme cytochrome MtrC, Fig. 1*A*, from *Shewanella oneidensis* MR-1. In the native state, MtrC contains 10 optically indistinguishable His/His-ligated hemes (27, 28). For this study, we aimed to introduce a spectrally distinct heme within the MtrC heme chain noting that His/Met hemes in their Fe(II) states have a broader, red-shifted Soret band than their His/His-ligated counterparts (29). Thus, we introduced to MtrC a methionine in place of the distal His561 ligand of Heme 8, Fig. 1*A*, blue. In addition, we replaced Tyr657 of MtrC, Fig. 1*A*, yellow, with cysteine to allow site-selective attachment of an ET phototrigger adjacent to Heme 10, a terminus of the heme chain. For our experiments, and as described in *SI Appendix*, Y657C H561M MtrC was labeled with the thiol-reactive photosensitizer dye [Ru(4-bromomethyl-4’-methylbipyridine)(2,2’-bipyridine)_2_](PF_6_)_2_ that has been previously well characterized (2, 30–33). We refer to the resulting protein as Ru-MtrC Met8. For that protein, any reduction of Heme 8 that is detected following excitation of the Ru-dye and ET into Heme 10 would report on ET across three hemes, Fig. 1*B*. We were unable to prepare diffracting crystals of Ru-MtrC Met8 or Y657C H561M MtrC. However, diffracting crystals of H561M MtrC could be obtained under similar conditions as for wild-type MtrC (27), and the structure, resolved to 1.60 Å, confirmed Met561 as the distal ligand of Heme 8 with a Fe-S(Met) distance of 2.3 Å, *SI Appendix*, Fig. S2. Superposition of the wild-type and mutant protein structures revealed no major global structural differences, with a total main chain rmsd of 0.3 Å as calculated using SUPERPOSE (34). The rmsd of both main chain and sidechain atoms within 6 Å of hemes 8, 9, and 10 (minus the distal ligand of heme 8) was also calculated, yielding values of 0.2 Å and 0.4 Å, respectively, indicating no significant localized changes surrounding these hemes.

**Fig. 2.**
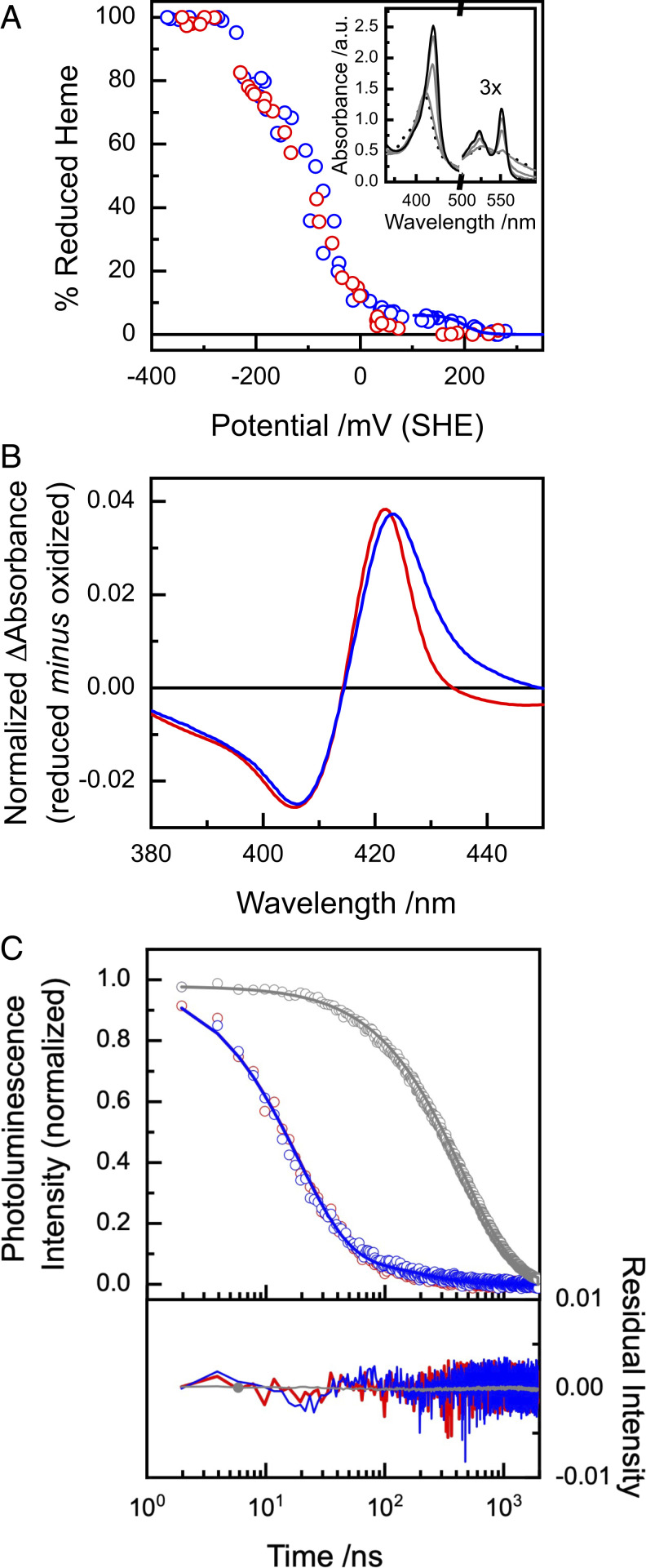
Redox and spectral properties of Ru-MtrC Met8 and Ru-MtrC His8. (*A*) Extent of reduction (circles) for Ru-MtrC Met8 (blue) and Ru-MtrC His8 (red) as determined by optically monitored potentiometric solution titration. Nernstian behavior (blue line) for single *n* = 1 center with *E*_m_ = 199 mV. (*Inset*) Spectra of Ru-MtrC Met8 at potentials of +253, +64, −86, −184, and −273 mV versus SHE where absorbance units = a.u. (*B*) Ultraviolet-visible absorbance difference spectra (reduced minus oxidized) of His/His (red) and His/Met (blue) ligated MtrC hemes, see *Results* for details. (*C*) Photoluminescence decay for Ru-MtrC Met8 (blue), Ru-MtrC His8 (red), and [Ru(II)(4-bromomethyl-4,4’-methylbipyridine)(bipyridine)_2_]^2+^ (gray). Excitation at 485 nm (500 kHz) with emission measured at 625 nm. Data for Ru-MtrC presented after removal of the contribution of MtrC alone, see *SI Appendix* for details. Fits (lines) have the parameters of Table 1 for Ru-MtrC proteins and a single exponential decay for the Ru-dye (*k* = 2 × 10^6^ s^−1^). As the fitting results for Ru-MtrC Met8 and Ru-MtrC His8 are almost the same, the red line (His8) is obscured by the blue line (Met8). All measurements in anaerobic 20 mM Tris HCl, 100 mM NaCl, pH 8.5.

To allow the properties of Ru-MtrC Met8 to be compared to those of the protein with only His/His-ligated heme, a second MtrC variant with a single substitution, Y657C, was prepared. When labeled with the aforementioned photosensitizing Ru-dye, we refer to that protein as Ru-MtrC His8. Redox properties of the two Ru-MtrC proteins in solution were compared through optically monitored potentiometric titration, Fig. 2*A*. A high-potential center (*E*_m_ +199 ± 16 mV versus standard hydrogen electrode [SHE], error is SEM) was revealed for Ru-MtrC Met8 for which there was no counterpart in Ru-MtrC His8. Similar behavior was noted in protein film cyclic voltammetry, *SI Appendix*, Fig. S5. The high-potential center was confirmed as His/Met-ligated Heme 8 by spectral analysis, *SI Appendix*, Fig. S4*B*, of the Met to Fe(III) charge transfer band at 695 nm (29), which disappeared on incubation of Ru-MtrC Met8 with the mild-reductant sodium ascorbate. Significantly, for this study, the much more intense peak at Soret wavelengths (380 to 450 nm) readily distinguished reduction of His/Met from His/His-ligated MtrC Fe(III) hemes. For these wavelengths, the difference spectrum (reduced minus oxidized heme), Fig. 2*B*, had a positive feature that was noticeably broader and red shifted for His/Met heme than the corresponding feature arising from His/His-ligated heme. As we illustrate in the following paragraphs, these properties allowed pump-probe spectroscopy to distinguish between Fe(II) hemes with different ligand sets.

**Fig. 3.**
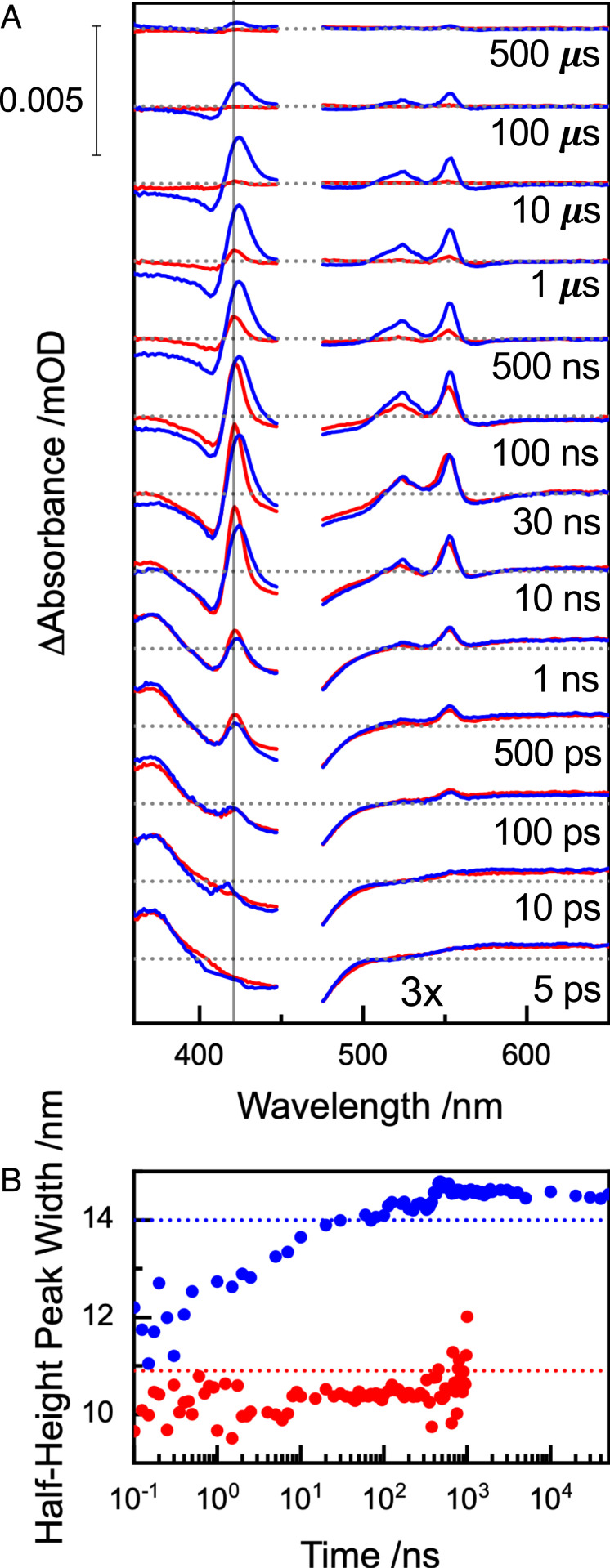
Transient absorbance of Ru-MtrC Met8 (blue) and Ru-MtrC His8 (red). (*A*) Differential absorbance spectra (post- minus preexcitation) for the indicated times after irradiation. Pulsed irradiation was at 457 nm and contributions from electronically excited hemes are removed, see *SI Appendix* for details. Samples contained Ru-MtrC (5 μM for measurements <440 nm, ∼150 μM for measurements >470 nm) in anaerobic 20 mM Tris HCl, 100 mM NaCl, pH 8.5. Spectra are presented with mOD corresponding to 5 μM protein. (*B*) Half-height width (circles) of the positive feature at Soret wavelengths for the indicated times after irradiation. Values indicative of MtrC His/Met heme (blue dashed line) and His/His heme (red dashed line) are from Fig. 2*B*.

Time-resolved photoluminescence spectroscopy provided preliminary evidence for ET from the photoexcited Ru-dye (^3^Ru) to Fe(III)-containing heme of the MtrC proteins, Fig. 1 *B*, ③. In the absence of quenching mechanisms, ^3^Ru decays by photoluminescence, Fig. 1 *B*, ②, with a lifetime of ∼450 ns (20). ^3^Ru is a metal-to-ligand charge transfer state as confirmed previously spectroscopically and by quantum chemical calculations showing that the triplet highest occupied molecular orbital (HOMO) is delocalized mainly over the bipyridine ligands (20). Significantly faster photoluminescence decays indicative of quenching by ET were displayed by ^3^Ru bound to the MtrC proteins containing 10 Fe(III) hemes, Fig. 2*C*. For both proteins, the data were well described as biexponential decays attributed (20) to the presence of multiple Ru-dye conformers, unable to exchange on the experimental timescale and having different charge separation rates. The conformer contributions and rate constants for ^3^Ru to Fe(III) charge separation, Fig. 1 *B*, ③, needed to describe the photoluminescence decays of both Ru-MtrC proteins, were very similar, Table 1. Thus, the Ru-dye experiences a comparable environment on the surface of both proteins with no detectable influence from the ligation of Heme 8.

**Table 1.** Photoluminescence decay properties* of Ru-MtrC proteins

	Ru-MtrC			
	Met8	His8	Met8	His8
Conformer	*a*	*a*	*b*	*b*
Contribution (%)	9 ± 3	11 ± 0.4	91 ± 2	89 ± 1
*k*_cs_^†^ (/10^6^ s^−1^)	5 ± 0.1	8 ± 0.1	55 ± 1	56 ± 1

* Errors are SD.

^†^ *k*_cs_ = 1/(decay lifetime, τ).

Direct evidence for ^3^Ru quenching by ET to MtrC ferriheme was provided by pump-probe spectroscopy. Metal-to-ligand charge transfer of the Ru(II)-dye was induced by laser excitation at 457 nm, as previously described (20). The time-resolved transient absorbance of the Ru-MtrC proteins arising solely from Ru(II)-dye excitation, Fig. 1*B*, is presented in Fig. 3*A*. The presented spectra have had the heme excited-state dynamics subtracted such that negative features arise from transiently depleted populations, for example, Ru(II)-dye ground state and Fe(III) heme. The positive features arise from transiently increased populations, for example, ^3^Ru and Fe(II) heme. Full details of data collection and analysis are provided in *SI Appendix*.

Immediately after excitation, the peak detected at 370 nm comes from π→π* transitions in the anionic ligand of the ^3^Ru state that is formally [Ru(III)(bipyridine)_2_(bipyridine^−^)] (35–37). Over time, disappearance of the ^3^Ru peak is accompanied by the simultaneous appearance of positive features centered on 552 and 522 nm that correspond to the α- and β-bands, respectively, of reduced Fe(II) hemes, for example, Fig.2 *A*, *Inset*. ^3^Ru quenching through electron injection into the heme chain is also evident in the appearance of a bisignate feature in the Soret region (380 to 450 nm). Significantly, the positive lobe of this feature from Ru-MtrC Met8 broadens over time, Fig. 3*B*, blue. At short times, the width is typical of His/His Fe(II) heme. By 4 μs, the width has increased to a value typical of His/Met Fe(II) heme. A red shift of the maximum for the corresponding peak over similar times, Fig. 3*A*, blue, confirms ET to His/Met-ligated Heme 8.

The transient absorbance of Ru-MtrC His8 in the Soret region, Fig. 3*A*, red, describes reduction of only His/His ligated hemes as expected. The positive lobe of the corresponding feature always has a half-height width typical of His/His-ligated Fe(II) heme, Fig. 3*B*, red. For both proteins, the transient absorbance spectra at longer times describe return to the ground state by charge recombination through ET from ferroheme to Ru^3+^, Fig. 1 *B*, ④. Depletion of the ground-state Ru-dye population is evident in the broad trough between ∼400 and 500 nm, and this feature recovers over time as the system returns to the ground state (*SI Appendix*, Fig. S7 for transient spectrum of Ru-dye only). A striking feature of the data is that ground-state recovery occurs more slowly for Ru-MtrC Met8 than Ru-MtrC His8, and we return to consider this aspect of the behavior in the *Discussion*.

**Scheme 1.**
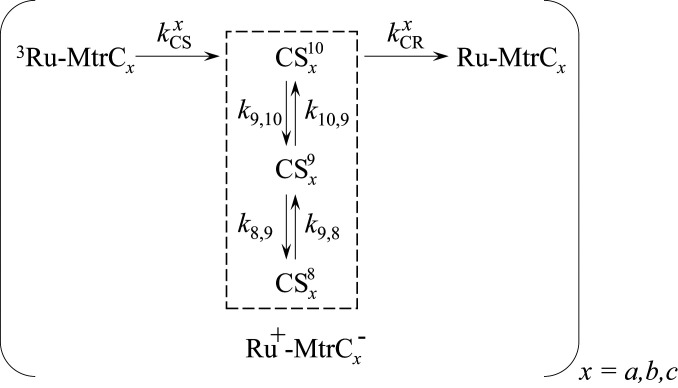
Photocycles operating in the Ru-MtrC proteins with three kinetically distinct conformers, *x* = *a*, *b*, and *c*.

To describe the photocycles operating in the Ru-MtrC proteins, Scheme 1 recognizes that charge separation and charge recombination in the Ru-MtrC proteins are strongly exergonic, Fig. 1*B*, and hence irreversible processes. Contributions from three kinetically distinct conformers of the protein, *x* = *a*, *b*, and *c* are justified by analysis of the transient absorbance, as described in the following paragraphs. ET between the Ru-dye and MtrC is assumed to occur only at Heme 10 since the distance between the bipyridine edge of the Ru-dye and the heme edge of nearby Heme 9 and Heme 8 is significantly longer than that to Heme 10, for example, 14, 18, and 5 Å, respectively. The distances are obtained from molecular dynamics (MD) simulation of Ru-MtrC His8 and the conclusion relevant to all conformers, reference *SI Appendix* for simulation details. Assuming an exponential distance decay constant of 1.39 Å^-1^ (38) and keeping all other ET parameters the same, the electron injection rates from Ru-dye to Heme 9 and 8 are estimated to be a factor of 10^5^ and 10^7^ smaller than for Heme 10 justifying this assertion. Electron migration along the heme chain in the Ru^+^-MtrC^−^ charge separated states is then included as the $CS_{x}^{10}$ ↔ $CS_{x}^{9}$ and $CS_{x}^{9}$ ↔ $CS_{x}^{8}$ interconversions where the electron resides on Heme *i* in $CS_{x}^{i}$, and *k*_*i*-1,*i*_ describes the rate constant for Heme *i* → Heme *i*-1 ET.

Scheme 1 is based on the spectroscopic observation that Heme 8 is reduced in Ru-MtrC Met8. We assume the same behavior in Ru-MtrC His8 and justify this by noting that the $CS_{x}^{10}$ ↔ $CS_{x}^{9}$ ET kinetics are expected to be the same in both proteins, that is, independent of the distal ligand to Heme 8, which as we see below is consistent with our results. Moreover, the Heme 9 → 8 ET in MtrC His8 is predicted (21) to be only slightly endergonic (0.1 eV) and have a similar rate to Heme 10 → 9 ET such that both reversible ETs should be included in modeling the Ru-MtrC His8 populations. We note that Scheme 1 considers only ET within Ru-MtrC monomers. Two observations show that intermolecular ET between proteins is negligible: the good agreement between the dynamics of the charge separated states at 5 and 150 µM protein, *SI Appendix*, Fig. S8, and solution masses of the (Ru-)MtrC proteins defined by analytical ultracentrifugation, *SI Appendix*, Fig. S3, that are close to those measured for the corresponding monomer by liquid chromatography–mass spectrometry.

**Fig. 4.**
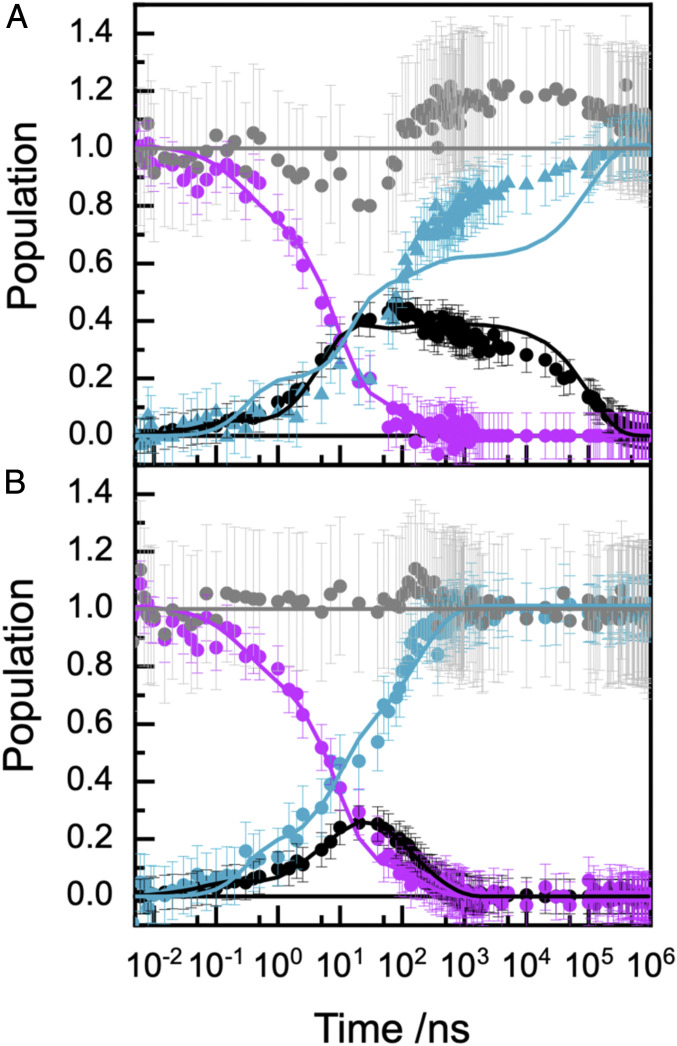
Progress of the Ru-MtrC Met8 (*A*) and Ru-MtrC His8 (*B*) photocycles. Evolution of the experimentally defined populations (circles) of ^3^Ru (purple) and Ru^+^-MtrC^−^ (black) together with recovery of Ru-MtrC (cyan) and the sum of these concentrations (gray). Fit (lines) to Scheme 1 with the parameters of Tables 2 and 3. The population of ^3^Ru at 5 ps was defined as 100%.

The prominent spectral features of the pump-probe spectroscopy, Fig. 3*A*, were used to define the transient populations of the species in Scheme 1 and in turn the ET kinetics. The ^3^Ru state was quantified at 370 nm. Reduced Fe(II) heme was quantified by fitting a single Gaussian to the sharp Q-band feature centered at 552 nm as a measure of the charge separated state, Ru^+^-MtrC^−^. Recovery of the ground-state Ru-dye was quantified through the bleach at 475 nm. Full details of this analysis are provided in *SI Appendix*, and the outcomes are presented in Fig. 4. Importantly, no additional species are needed to describe the photocycle as the transient populations are accounted for throughout. We note that for Ru-MtrC Met8 the sum of populations is slightly greater than 1 between 100 ns and 100 μs, Fig. 4*A*. This is most likely due to an overestimation of the Ru(II)-dye ground-state population at these times when the charge separated state (Ru^+^-MtrC^−^) is at a maximum and absorbance of the Ru(III)-dye associated with the long-lived charged separated state, Fig. 1*B*, will make a small positive contribution to the transient at 475 nm (39) where the ground-state Ru(II) population was quantified as a negative contribution (bleach). However, contributions from absorbance by the Ru(III)-dye do not impact on our quantification of the populations of ^3^Ru and Fe(II) heme from which the kinetic parameters are derived, as described in the following paragraphs.

Excellent descriptions of the measured ^3^Ru and Ru^+^-MtrC^−^ populations for both Ru-MtrC proteins, Fig. 4, are provided by Scheme 1 with the conformer contributions and rate constants of Tables 2 and 3. The best fit results for Ru^+^-MtrC^−^, which is the most certain experimentally defined population due to highest signal to noise, are shown (*R*^2^ = 0.966 for Ru-MtrC Met8; *R*^2^ = 0.965 for Ru-MtrC His8). We note that in using Scheme 1 to describe the measured transients, the fit parameters were kept to a minimum. The heme–heme ET rate constants are taken to be the same for all conformers of a given protein because heme–heme electronic coupling is a short-range effect and unlikely to be affected by the position of the Ru-dye. In addition, minimal differences in free energies (*E*_m_ and reorganization energy [λ]) are expected (40) because of the relatively large separation of the hemes from the label (>5 Å). The parameters describing initial charge separation (*k*_CS_ and % contributions) were taken to be the same for both proteins on the basis of the time-resolved photoluminescence. The number of conformers undergoing charge separation, Fig. 1 *B*, ③, was defined by the decay of the ^3^Ru populations, Fig. 4, purple, and *SI Appendix*, Fig. S10. From 1 to 1,000 ns after excitation, the decay was biexponential for both proteins and described by *k*_cs_ values, Table 2, in agreement with those from time-resolved photoluminescence spectroscopy, Table 1. At shorter times, the transient absorbance of both Ru-MtrC proteins revealed a third short-lived exponentially decaying contribution from an additional conformer displaying faster charge separation that was not resolved by time-resolved photoluminescence. As a consequence, contributions from three conformers were included when using Scheme 1 to describe the measured transients.

**Table 2.** Conformer contributions and rate constants for charge separation and charge recombination from transient absorbance of Ru-MtrC proteins

	Conformer		
	*a*	*b*	*c*
Contribution* (%)	14	66	20
*k*_CS_* (/10^6^ s^−1^)	5.2	112	3,840
*k*_CR_ Ru-MtrC His8 (/10^6^ s^−1^)	71	301	11,100
*k*_CR_ Ru-MtrC Met8 (/10^6^ s^−1^)	333	63	108,000

* Values derived from fit of experimental data from Ru-MtrC Met8 and His8 as described in the Results and *SI Appendix*.

**Table 3.** Heme-to-Heme ET rate constants*

	Rate constant^†^ experiment (theory)	
	Ru-MtrC Met8	Ru-MtrC His8
*k*_9,10_ (/10^6^ s^−1^)	76 (300)^‡^	219 (300)^¶^
*k*_10,9_ (/10^6^ s^−1^)	66 (130)^‡^	14 (130)^¶^
*k*_8,9_ (/10^6^ s^−1^)	11,100 (68,000)^§^	105 (18)^¶^
*k*_9,8_ (/10^6^ s^−1^)	3.3 (0.6)^§^	1,560 (1,940)^¶^

* Values derived from fit to experimental data (MD/DFT calculations), as described in the *Results* and *SI Appendix*.

^†^ *k*_9,10_, *k*_10,9_, *k*_8,9_, and *k*_9,8_ are defined in Scheme 1 and assumed to be the same for all conformers of each protein.

^‡^ Assumed to be the same as for Ru-MtrC His8.

^§^ Obtained from the nonadiabatic (Marcus) rate equation using computed parameters for electronic coupling, reorganization free energy, and driving force. See *SI Appendix* for details of the calculations.

¶ Jiang et al. (21). The present values differ very slightly from the values in ref. 21 due to a minor error in the electronic coupling calculations there, which are now corrected in this manuscript.

Possible molecular models of the three conformers were explored by docking the Ru-dye to Tyr657 of MtrC using the X-ray structure for the latter (*SI Appendix*, Fig. S11). Molecular dynamics simulations of the lowest energy docking structures in aqueous solution (200 ns in total) revealed two long-lived conformers of the Ru-dye (*SI Appendix*, Fig. S12) that differ in their orientation with respect to Heme 10 (*SI Appendix*, Fig. S13). The average distance between the bipyridine and Heme 10 edges is short, 5 to 6 Å. Hence, we assign these conformers to the two kinetically distinct conformers *b* and *c* that exhibit fast injection kinetics. We also observe Ru-dye conformers that exhibit larger distances, 8 to 9 Å, which we assign to the slowest kinetic conformer *a.* Further details on the docking and MD simulations are given in *SI Appendix*.

**Fig. 5.**
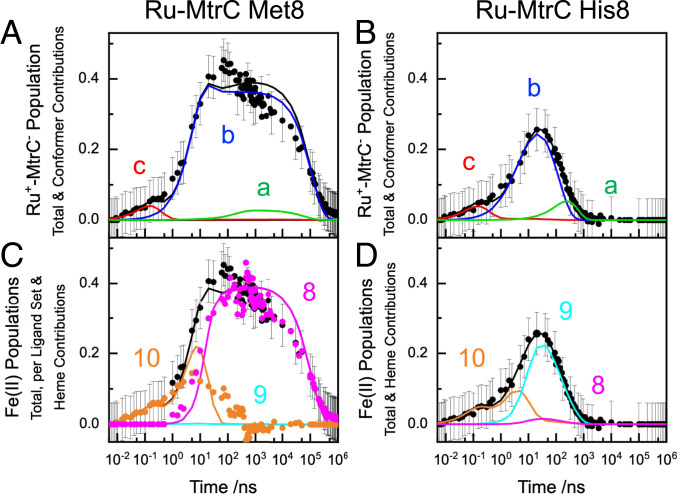
Contributions to the Fe(II)-containing Ru^+^-MtrC^−^ charge separated states for Ru-MtrC Met8 and Ru-MtrC His8. (*A* and *B*) Modeled contributions (lines) from conformers *a* (green), *b* (blue), and *c* (red) and their sum (black) for Ru-MtrC Met8 (*A*) and His8 (*B*) with the experimentally defined populations of Ru^+^-MtrC^−^ (black circles). (*C* and *D*) Modeled Fe(II) populations (lines) for Heme 10 (orange), Heme 9 (cyan), and Heme 8 (pink) and their sum (black). Experimentally defined populations (circles) of Ru^+^-MtrC^−^ (black) and for Ru-MtrC Met8 Fe(II) His/Met Heme 8 (pink) and Fe(II) His/His ligated Hemes 10 and 9 (orange), see *Results* for details. Modeled contributions are from Scheme 1 with the parameters of Tables 2 and 3.

The kinetic models were interrogated to better understand the observed behaviors. For both Ru-MtrC proteins, the majority of the charge separated state, Fig. 5 *A* and *B*, blue, arises from a single conformer, *b*, for which heme–heme ET is competitive with charge recombination. We note that the rate constants presented in Tables 2 and 3 were obtained from fitting the measured ^3^Ru and Ru^+^-MtrC^−^ populations, where the latter is the total amount of Fe(II) heme with no distinction between hemes having His/His and His/Met ligation. To validate our predicted rate constants for MtrC Met8, we therefore took advantage of the spectroscopically defined reduction of Heme 8 in that protein and compared the measured and predicted transient populations of reduced His/His- and His/Met-ligated hemes, Fig. 5 *C* and *D*. For Ru-MtrC Met8, the modeled Fe(II) Heme 8 population, Fig. 5*C*, pink line, is in good agreement with that deduced, pink circles, directly from the time-dependent increase in the Soret peak width, Fig. 3*B*. Charge separated states with the electron residing on Heme 10 (and 9) predominate immediately after excitation, and their total population is again in good agreement with that predicted by the model, Fig. 5*C*. Thus, our quantitative description of ET in Ru-MtrC Met8 is supported by the good agreement between the modeled and experimentally defined Fe(II) populations for the His/His- and His/Met-ligated hemes.

For Ru-MtrC His8, the predicted Fe(II) populations of Hemes 10, 9, and 8 are compared to the total Fe(II) population in Fig. 5*D*. The electron is predicted to reside on Heme 10 immediately after excitation and then migrate to Heme 9 with very little transfer to Heme 8. Experimental validation of these populations is precluded by the optical similarity of the hemes. However, inspection of the heme–heme ET rate constants indicates that they are realistic. A value for *k*_8,9_ that is 100× higher in Ru-MtrC Met8 than Ru-MtrC His8 is readily attributed to the lower reduction potential of Heme 8 with His/His than His/Met ligation. By contrast, the values of *k*_9,10_ are comparable (within 3×) for the two proteins where the reduction potentials of the corresponding hemes are expected to be similar. Moreover, for MtrC His8 *k*_8,9_ and *k*_9,10_ are comparable (within 2×) as anticipated given their similar driving forces (0.1 eV) (21).

## Discussion

**Fig. 6.**
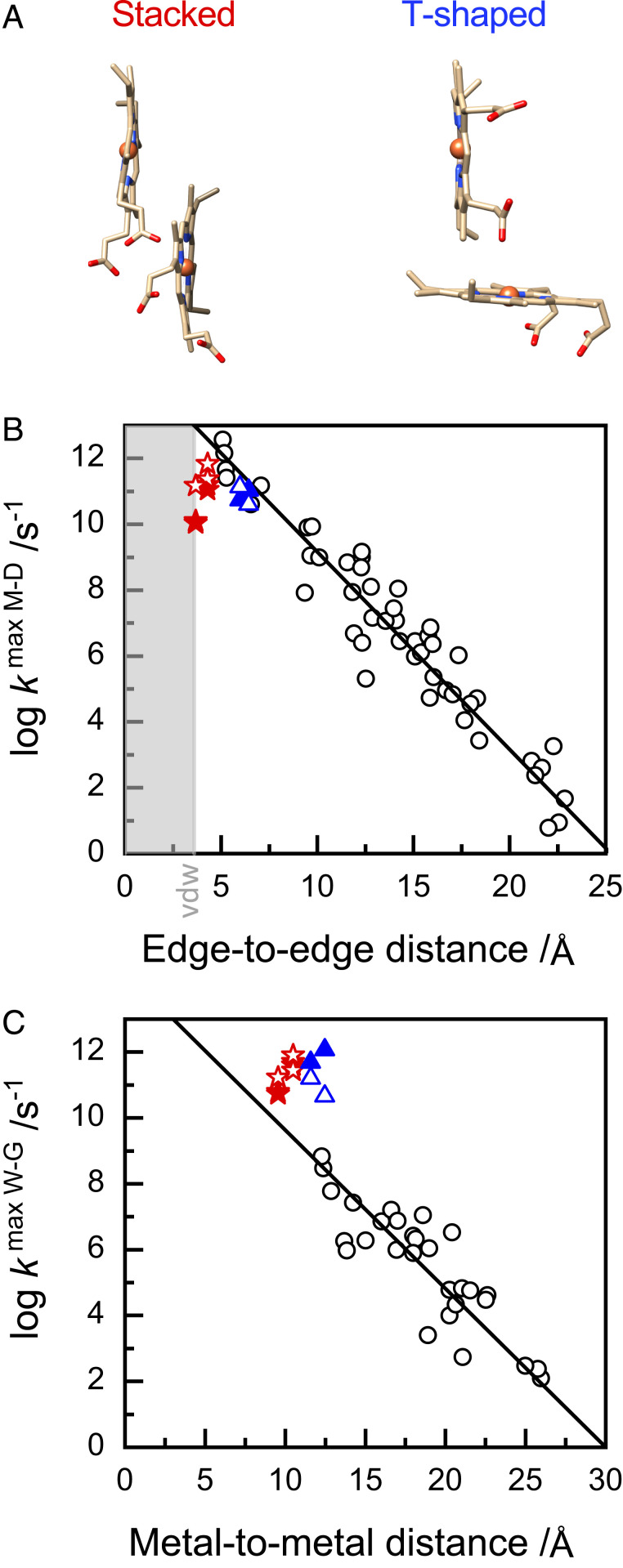
(*A*) Two heme-packing motifs frequently found in multiheme cytochromes. (*B*) Log of free energy–optimized rates (*k*^max^ ^M-D^) as a function of edge-to-edge distance (donor to acceptor). The van der Waals (vdw) contact distance is indicated. Values of λ used to calculate *k*^max^ ^M-D^ were as follows: 0.7 and 0.73 eV for MtrC heme pairs 10 ↔ 9 and 9 ↔ 8, respectively, and 1.08 and 0.88 eV for STC heme pairs 1↔ 2 and 3 ↔ 4, respectively. (*C*) Log of free energy–optimized rates (*k*^max^ ^W-G^) as a function of metal-to-metal distance (donor to acceptor). In *B* the Hopfield rate and in *C* the Marcus rate expressions were used to convert the experimental ET rates to free energy-optimized ET rates. For *B* and *C*, values from experiment (filled) and computation (open) are for stacked heme pairs (red stars), this work, and T-shaped heme pairs (blue triangles) from previous work (20, 42). Values for other natural and engineered redox proteins (gray circles) and the M-DR and W-GR (lines): for *B* are as detailed in ref. 3. Copyright (2010), with permission from Elsevier, and for *C* are as detailed and reproduced from ref. 23 with permission from ACS, further permissions related to the material excerpted should be directed to the ACS.

Introducing a spectrally unique His/Met ligated heme at a site distant from a Ru-photosensitizer has allowed unambiguous detection of intramolecular ET within the MtrC heme chain. Transient absorbance has quantified rate constants for ET between the intervening heme pairs. The values, summarized in Table 3, describe ET between Hemes 10 ↔ 9 and Hemes 9 ↔ 8 with closest edge–edge distances of 3.7 and 4.3 Å, respectively. Both ET events occur between hemes with a stacked configuration, Fig. 6 *A*, *Left*, where the porphyrin ring planes are parallel and overlap to place the rings in van der Waals contact. Such configurations are prevalent in multiheme cytochromes alongside the T-shaped heme pairs, Fig. 6 *A*, *Right*, for which we previously (20) proposed ET rate constants from transient absorbance of the STC tetraheme cytochrome photosensitized with the Ru-dye used in this study.

When comparing biological electron tunneling in different proteins, it is customary to remove all the effects that come from driving force and treat the tunneling as activationless. In accord with the Moser–Dutton (M-D) treatment, we use the Hopfield one-mode quantized ET rate expression to convert the measured ET rates (in the direction where *ΔG* < 0) to the free energy–optimized ET rates by multiplying a term $10^{\left(3.1{\left(\Delta G+\;\lambda \right)}^{2}/\lambda \right)}$ with the measured ET rates (41). The *ΔG* values are taken from the ratio of measured forward and backward ET rate constants, and the values for λ are taken from computation (21, 42) and provided in the legend to Fig. 6. The resultant experimental free energy–optimized ET rates, denoted *k*^max^ ^M-D^, are shown in Fig. 6*B* versus edge-to-edge distance (full symbols), together with the computed free energy–optimized Hopfield ET rates (empty symbols). Edge-to-edge distance refers to the smallest distance of any pair of atoms where one atom belongs to the heme macrocycle of the donor heme and the other atom to heme macrocycle of the acceptor heme. It is immediately apparent that the ET between the stacked and the T-shaped heme pairs of multiheme cytochromes display some of the highest reported free energy–optimized ET rate values: for stacked hemes, the rates are 11 − 156 × 10^9^ s^−1^ (145 − 682 × 10^9^ s^−1^ from computation), and for T-shaped hemes, 55 − 107 × 10^9^ s^−1^ (40 − 139 × 10^9^ s^−1^ from computation). Indeed, those free energy–optimized values are not much slower than the initial rapid events on the picosecond time scale that drive charge separation in photosynthetic reaction centers (though, the actual, thermally activated heme–heme rates are of course three orders of magnitude smaller). Interestingly, there is precedent for such high rates in cytochromes. Very similar *k*^max^ values are obtained for the heme *a* to heme *a*_3_ ET reaction, 21 × 10^9^ s^-1^, in the proton pump cytochrome *c* oxidase (43–45) (T-shaped motif) using the ET parameters of ref. 46.

Our data for the MtrC stacked heme pairs fall two to three orders of magnitude below the M-DR, shown by the line of Fig. 6*B*, when comparing edge-to-edge distances. By contrast, free energy–optimized ET rates, denoted *k*^max^ ^W-G^, calculated by the method of Winkler and Gray (23) are two to three orders of magnitude higher, Fig. 6*C*, than the Winkler–Gray ruler (W-GR) when comparing metal-to-metal distances. These discrepancies could be due to one or more of the following reasons. First, there is a lack of measured rate constants for very short-ranged ground-state ET in biology, hence the M-DR and W-GR rulers have a larger uncertainty here than for larger distances. Second, the notion that a simple distance metric such as metal-to-metal or edge-to-edge distance is a good descriptor for short-range ET can be debated. At short distances, the frontier orbitals of electron donor and acceptor interact and overlap directly—these orbitals have a complicated nodal shape, and it is the relative orientation of the two cofactors, in addition to distance, that determines the electronic coupling and hence the *k*^max^ values. Third, tunneling between very closely packed cofactors is typically through space (as, e.g., is the case of the stacked heme pairs 10/9 and 9/8), not through a protein medium, as for most of the other redox partners included in the M-DR and W-GR plots. The barrier for through-space tunneling is higher and leads to lower tunneling rates than what one would expect based on the M-DR. By contrast, the *k*^max^ ^M-D^ values for the T-shaped heme pairs of STC are very well described by the M-DR. In that case, tunneling is mediated (42) by the cysteine linkages inserting in the space between the hemes in a scenario that is closer to the through-protein tunneling assumed by the M-DR.

The MtrC His8 Heme 10↔9 and Heme 9↔8 ET rates predicted previously (21) by density functional theory (DFT) and MD calculations are in excellent agreement with experiment; the deviation is, at most, a factor of six, Table 3. This is notable for two reasons: First, all ET parameters were computed with general state-of-the-art methodologies (i.e., not tailored/tuned toward the specific system under investigation). Second, the calculations were carried out before the present experimental data were obtained. This gives confidence in the set of heme–heme ET rates that we have recently predicted for a number of multiheme cytochromes using the same computational methodology and for which there are no experimental rate data available yet. According to those calculations, similarly high rates in the (1 to 10 ns)^-1^ range can be expected for most stacked heme pairs in related multiheme cytochromes of *Shewanella*, MtrA (22), MtrF (21), and STC (42), as well as in the hexaheme cytochrome of *Geobacter*, OmcS (22). These calculations also showed that the maximum possible, that is, protein-limited, electron flux through these proteins is significantly smaller than the nanosecond rates between stacked pairs. That flux is typically limited by the T-shaped heme pairs to about 10^5^ – 10^6^ s^−1^ in MtrC and all the above proteins. The highest flux measured to date (47) through the 20 heme wire of the MTR complex defined by the MtrC and MtrA cytochromes is ∼1 × 10^4^ s^−1^ and most likely limited by heterogeneous ET from the protein to the Fe(III)-oxide particles. This is in agreement with a recent computed estimate of 3 × 10^4^ s^−1^ (22).

*Shewanella* MtrC has evolved to support anaerobic respiration, specifically to deliver electrons from intracellular respiratory chain enzymes to extracellular terminal electron acceptors such as mineral oxides of Fe(III) and Mn(IV) and humic substances (10). Net electron flux for these pathways measured in laboratory experiments is of the order of 1 s^−1^ MtrC^−1^ (48–50) such that there initially appears to be no need for these proteins to have evolved structures supporting much faster electron fluxes. However, securing respiratory integrity in the dynamically changing, chemically stratified soils and sediments is likely to represent a different challenge. In such situations, opportunities to discharge respiratory electrons to extracellular electron acceptors are likely to be fleeting. Multiheme cytochromes, including polymerized cytochrome wires such as *Geobacter* OmcS (8, 9), with their numerous redox centers provide the opportunity to store respiratory electrons until there is opportunity to discharge them when an extracellular electron acceptor comes close enough. When this situation arises, rapid ET across the bacterial network of multiheme cytochromes will ensure that such encounters, regardless of precise location, allow ET to the acceptor. Very rapid intracytochrome ET is also likely to benefit the long-distance ET, over several micrometers, which occurs across *S. oneidensis* outer membrane extensions of both planktonic cells and electrode supported biofilms. In this process MtrC is critical, and recent studies (51, 52) suggest a “collision-exchange” process whereby long-distance ET is facilitated by electron exchange between cytochromes that diffuse laterally across the membrane surface. The rapid intracytochrome ET described in this study enhances the probability of intercytochrome electron exchange in the encounter complexes.

Another feature of extracellular cytochromes may facilitate their contributions to anaerobic respiration, namely the positioning of many hemes within 10 Å of the protein surface. This architecture will facilitate ET to acceptors that engage with numerous, spatially distinct sites on that surface. This is in contrast to most oxidoreductases for which Moser and Dutton noted (3) the majority of redox sites are buried within insulating protein to keep them away from most substrates and achieve selectivity of redox transformations at buried active sites. Indeed, it was argued (45) that the fast nanosecond rate for heme *a* to *a*_3_ ET in cytochrome *c* oxidase could ensure that heme *a*_3_ is reduced in the infrequent event of oxygen binding (oxygen does not bind to the oxidized form of heme *a*_3_). The functional role of the high-ET rates in both extracellular and intracellular respiration could then represent distinct evolutionary strategies to maximize the chances for successful trapping and reduction of the corresponding terminal electron acceptors. Intracellular respiration aims to minimize the production of toxins such as reactive oxygen or nitrogen species that can rapidly damage DNA etc. When the terminal electron acceptor is reduced outside the organism, the cell wall offers protection against toxicity such that there is less need for intrinsic control over the products of reduction.

In closing, we note that in addition to affording a viable route to quantifying heme-to-heme ET rates in multiheme cytochromes, our work demonstrates that the charge separated state of our photosensitized protein is significantly stabilized by introduction of a His/Met-ligated heme. This observation, which can be attributed to the creation of a site of more positive reduction potential providing a strong thermodynamic sink within the heme wire, has important implications for the design of biohybrid materials that can effectively couple single-photon light-driven ET to multielectron catalysis to mimic the photosynthetic paradigm. The intrinsic charge storage capacities of the heme wires found within multiheme cytochromes, such as the decaheme MtrC, makes them attractive components for materials that feature rapid unidirectional ET from photosensitizers through charge accumulation modules to redox-active catalytic sites. We have shown here that locating a Ru(II)-dye photosensitizer at MtrC residue 657 ensures heme–heme ET is competitive with charge recombination in the majority of conformers. Furthermore, the probability of subsequent charge accumulation is increased when His/His-ligated heme is replaced by its His/Met-ligated counterpart, such that the charge separated state persists to beyond 100 μs. Thus, our work illustrates guiding principles that can be developed to engineer multiheme cytochromes as components of versatile photosynthetic biohybrid assemblies.

## Materials and Methods

### Protein Preparation and Characterization.

Ru-MtrC Met8, Ru-MtrC His8, and H561M MtrC were prepared using previously explained methods (53, 54) and described in detail in *SI Appendix*. Potentiometric titration as described in ref. 55 and time-resolved photoluminescence spectroscopy were performed in anaerobic 20 mM Tris HCl, 100 mM NaCl, pH 8.5 with ∼1 μM protein. Sedimentation equilibrium ultracentrifugation was performed as described in ref. 55. Experiments were carried out with 0.4 µM protein in 50 mM Na_2_HPO_4_/NaH_2_PO_4_, 50 mM NaCl, and 0.1% (volume/volume) Triton X-100, pH 7.5. Data were analyzed using Ultrascan II (56). Further details of all these methods, protein film voltammetry, and the associated data analyses are provided in *SI Appendix*. Details of protein crystallization, X-ray diffraction data collection, and structure determination of H561M MtrC are provided in *SI Appendix*.

### Transient Absorbance Spectroscopy.

Measurements were performed with anaerobic solutions containing 20 mM Tris HCl, 100 mM NaCl, pH 8.5 and in the absence of sacrificial redox partners. Prior to irradiation, all hemes were in the oxidized Fe(III) state. Time-Resolved Multiple Probe Spectroscopy transient absorbance was performed at the Central Laser Facility of the Rutherford Appleton Laboratory using the apparatus described previously (57, 58). Data collection followed excitation at 457 nm as previously described (20). Further details of data collection together with full details of the data processing are provided in *SI Appendix*.

### Model Fitting for ET Dynamics.

The combined data of ^3^Ru-MtrC His8 and ^3^Ru-MtrC Met8 population decays were fit to rate constants $k_{CS}^{x}$ and % contribution for models comprised of different numbers of conformers *x* (*SI Appendix*, Eq. **S10**). A minimum of three conformers, *x* = *a*, *b*, *c* were required to fit the data (*SI Appendix*, Fig. S10*A*). This was followed by a fit of the Ru^+^-MtrC^−^ populations for each of the two Ru-MtrC proteins to obtain the rate constants $k_{10,9}$, $k_{9,10}$, $k_{8,9}$, $k_{9,8}$, and $k_{CR}^{x}$ (*SI Appendix*, Eqs. **S11**–**S14**). *R*^2^ values of the fits are summarized in *SI Appendix*, Table S10. Further details of the model fits are provided in *SI Appendix*.

### Atomistic Description of Heme–Heme ET in Ru-MtrC Met8.

The rate constants for ET between His/His Heme 9 and His/Met Heme 8 of the Ru-MtrC Met8 protein, *k*_8,9_ and *k*_9,8_, respectively, were obtained from the nonadiabatic (Marcus) rate equation. Electronic coupling between the hemes, reorganization free energy, and driving force were calculated as described in detail in *SI Appendix*.

## Supplementary Material

Supplementary File

## Data Availability

The H561M MtrC structure and the associated structure factors are deposited in the Protein Data Bank under the access code 7O7G. Datasets used to make figures are deposited at Figshare (DOI: 10.6084/m9.figshare.16622221).
