# Supplementary material for: Nanosecond heme-to-heme electron transfer rates in a multiheme cytochrome nanowire reported by a spectrally unique His/Met-ligated heme
Source: Proc Natl Acad Sci U S A. 2021 Sep 23;118(39):e2107939118. doi: 10.1073/pnas.2107939118 (PMC8488605; doi:10.1073/pnas.2107939118)
Supplement: Supplementary File [file pnas.2107939118.sapp.pdf]

Supplementary Information for

Nanosecond Heme-to-Heme Electron Transfer Rates in a Multiheme Cytochrome Nanowire Reported by a Spectrally Unique His/Met Ligated Heme

Jessica H. van Wonderen, Katrin Adamczyk, Xiaojing Wu, Xiuyun Jiang, Samuel E. H. Piper, Christopher R. Hall, Marcus J. Edwards, Thomas A. Clarke, Huijie Zhang, Lars J. C. Jeuken, Igor Sazanovich, Michael Towrie, Jochen Blumberger, Stephen R. Meech, Julea N. Butt.

Julea N. Butt
Email: j.butt@uea.ac.uk

Stephen R. Meech
Email: s.meech@uea.ac.uk

Jochen Blumberger
Email: j.blumberger@ucl.ac.uk

This PDF file includes:
Supplementary text
Figures S1 to S13
Tables S1 to S11
SI References

Contents

1. Protein Purification, Biochemical Analysis and Ru-dye Labeling 2
2. H561M MtrC Structure Determination 4
3. Analytical Ultracentrifugation 6
4. Time-Resolved and Static Photoluminescence Spectroscopy 6
5. Optically Monitored Potentiometric Titrations 7
6. Voltammetry 8
7. Quantification of Spectral Features in the Linear Spectra of the MtrC Proteins 9
8. Transient Absorbance Spectroscopy (TAS) 11
   8.1 Analysis of TAS Spectral Contributions 11
   8.2 Time-Dependent Concentration of Ru^{+}-$MtrC^{-}$ (Fe^{2+}) 13
   8.3 Time-Dependent Concentrations of Ru-MtrC and ^{3}Ru-MtrC 15
9. Fitting the transient populations 16
10. Computed rate constants $k_{8,9}$ and $k_{9,8}$ for Ru-MtrC Met8 18
11. Docking and molecular dynamics simulations of Ru-MtrC 19

1. Protein Purification, Biochemical Analysis and Ru-dye Labeling

Bacterial strains and plasmids used in this study are listed in Table S1 with primers (Eurofins) detailed in Table S2. Soluble MtrC with a C-terminal Strep II tag to assist purification is encoded by pJvW001 constructed from the pBAD202/D-TOPO vector (1). Y657 MtrC and H561M MtrC were prepared using PCR with the appropriate primers and pJvW001 as the template. The plasmid for Y657 MtrC served as the template for PCR based preparation of the Y657C H561M double mutant. The resulting plasmids were introduced into chemically competent *Escherichia coli* OneShot TOP10 cells, and the transformed cells were streaked onto lysogeny broth (LB) agar plates containing kanamycin (50 μg mL^{-1}). The purified plasmids were transformed by electroporation into *S. oneidensis* MR-1 and successful incorporation of the desired plasmids was confirmed by Sanger DNA sequencing (Eurofins).

Proteins were purified from spent media following arabinose induction of the corresponding cultures as previously described (1). LC-MS of the as purified Cys variants was performed as previously described (2) and revealed masses ≈100-300 Da higher than expected. LC-MS resolved single species with masses, Table S3, consistent with the expected peptide + 10 *c*-hemes after addition of the reducing agent tris(2-carboxyethyl)phosphine (TCEP) at 5 mM for 30 min, RT, followed by TCEP removal and exchange (3×) into 20 mM Tris-HCl, pH 7.5 using a micro-concentrator (5 kDa cut-off). It was concluded that the proteins were purified with a covalent attachment to the introduced cysteines, and that this was removed by TCEP-induced reduction cleavage. Extinction coefficients, Table S4, for the TCEP treated proteins were defined by pyridine-hemochrome analyses (3).

Cys-directed MtrC labeling with $[Ru(4\text{-bromomethyl-4'-methylbipyridine})(2,2'\text{-bipyridine})_2](PF_6)_2$ (HetCat, Switzerland) was performed as described previously (2) and confirmed by LCMS, Table S3. Labeling efficiencies, judged by UV-visible absorbance and LCMS, were close to 1:1 in the Ru-MtrC Met8 and Ru-MtrC His8 proteins. SDS-PAGE gels, Fig. S1, confirmed the purity of the samples used in this study. We note that in the non-reducing conditions of the SDS-PAGE of Fig. S1 the proteins carrying the Y657C mutation were resolved as monomers and dimers. This can be attributed to the presence of the Cys introduced to the protein surface. The higher molecular mass band is not present in reducing gels or after labeling the Cys residues with Ru-dye, Fig. S1. After exposing soluble MtrC with a C-terminal Strep II tag to equivalent conditions, there was no evidence of Ru-dye attachment. This confirmed labeling was of the Cys that replaced Tyr657 in Ru-MtrC His8 and Ru-MtrC Met8.

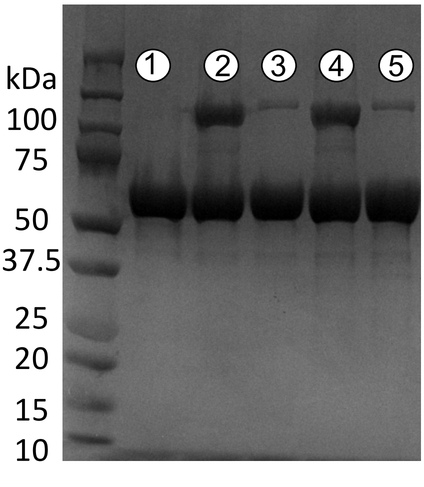

Fig. S1. SDS-PAGE gel of MtrC proteins ± Ru-dye. Lanes: (1) MtrC, (2) Y657C MtrC (MtrC His8) (3) Ru-Y657C MtrC (Ru-MtrC His8) (4) Y657C H561M MtrC (MtrC Met8) (5) Ru-Y657C H561M MtrC (Ru-MtrC Met8). Higher molecular weight bands (approx. 150 kDa) are protein Cys-Cys dimers. Left hand land: Molecular weight markers as indicated. Proteins visualized by Coomassie stain.

Table S1. Strains and Plasmids used in this Study.

	Relevant Feature	Source/Reference
**Strains**		
S. oneidensis		
MR-1	Wild type	Lab stock
JvW001	MR-1 containing pJvW001	(1)
JvW014	MR-1 containing pJvW014	This work
JvW015	MR-1 containing pJvW015	This work
JvW016	MR-1 containing pJvW016	This work
**Plasmids**		
pJvW001	pBAD/TOPO derivative encoding for WT MtrC^{CStrp}	(1)
pJvW014	pBAD/TOPO derivative encoding for Y657C MtrC^{CStrp} (His8)	This work
pJvW015	pBAD/TOPO derivative encoding for H561M Y657C MtrC^{CStrp} (Met8)	This work
pJvW016	pBAD/TOPO derivative encoding for H561M MtrC^{CStrp}	This work

Table S2. Primers used in this Study. Mutagenic codons underlined.

Primer	Sequence (5' → 3')	Description
Y657C Forward	CCTGTTTCTGCTGCCATAC	Introducing Y657C into MtrC^{CStrp}
Y657C Reverse	GTATGGCAGCAGAAACAGG	
H561M Y657C Forward	GAG CTA AAA CTA ATG AAA AAA CAC GTT	Introducing H561M into MtrC^{CStrp} Y657C
H561M Y657C Reverse	AAC GTG TTT TTT CAT TAG TTT TAG CTC	

Table S3. Summary of LCMS for Soluble MtrC Proteins with C-terminal Strep II tag.

	**MtrC His8**	**MtrC Met8**	**Ru-MtrC His8**	**Ru-MtrC Met8**
**Predicted Mass (Da)**	76 193	76 180	76 788	76 775
**Observed Mass (Da)**	76 200	76 188	76 787	76 782

Table S4. Pyridine Hemochrome Derived Extinction Coefficients.

	**Extinction Coefficient (mM^{-1} cm^{-1})**		**Difference Extinction Coefficient (reduced – oxidized) (mM^{-1} cm^{-1})**	
	**Oxidized Protein at 410 nm**	**Reduced Protein at 420 nm**	**421 – 405 nm**	**552 – 568 nm**
**MtrC**	1326 ± 14	1893	1708	228
**MtrC His8**	1389 ± 34	2015	1831	245
**MtrC Met8**	1363 ± 26	1847	1672	238

2. H561M MtrC Structure Determination

Prior to use in crystallization experiments, purified H561M MtrC was concentrated to ≈5 ml and applied to a Superdex 200 26/600 size-exclusion chromatography column equilibrated with 20 mM HEPES pH 7.8. Protein was eluted from the column at 1 ml min^{-1} and 2 ml fractions were collected. Fractions containing H561M MtrC were pooled and concentrated to ≈12 mg ml^{-1} utilizing a 30 kDa MWCO centrifugal concentrator.

H561M MtrC crystals were obtained from sitting-drop vapor diffusion crystallization at 277K with 0.4 M sodium acetate pH 4.5, 0.1 M $CaCl_2$ and 19% PEG 6,000 as the reservoir solution, similar conditions as to previously reported for MtrC (4). Crystals were obtained with both a 1:1 and 1:2 ratio of protein to reservoir solution with a total drop volume of 0.6 µl. Crystals were cryo-protected by transferring to a solution of 0.4 M sodium acetate pH 4.5, 0.1 M $CaCl_2$, 19% PEG 6,000 and 17% ethylene glycol before being vitrified by plunging into liquid nitrogen. Data to a final resolution of 1.60 Å were collected on MtrC crystals in a gaseous stream of nitrogen at 100 K on beamline I04 at Diamond Light Source (UK).

The structure was determined by molecular replacement with PHASER using the structure of *S. oneidensis* MR-1 MtrC (PDB ID 4LM8) as the search model. The final model was built through alternating rounds of model-building with Coot (5) and refinement with REFMAC (6). The final model was refined to an R_{work} (R_{free}) value of 17.0 (20.5) % with a single outlier in the Ramachandran plot. Structural superposition of MtrC and H561M MtrC performed with SUPERPOSE (7) revealed no major structural changes with an r.m.s.d of 0.3 Å. The only significant structural difference observed in the electron density is related to the distal ligand to Heme 8 that is resolved as Met in H561M MtrC, Fig. S2. Data collection and structure refinement statistics are provided in Table S5. Coordinates have been deposited in the RCSB Protein Data bank (PDB ID 7O7G).

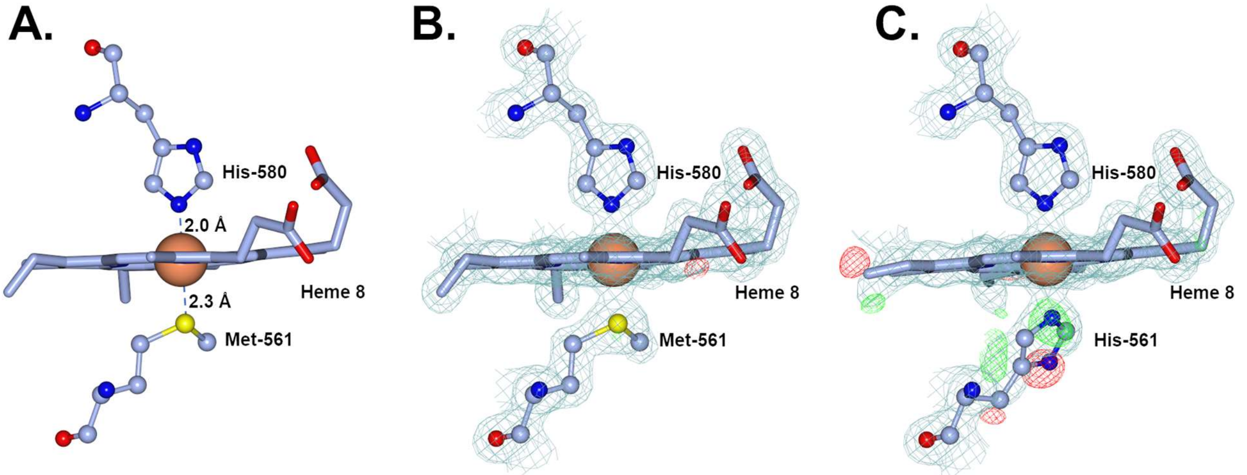

Fig. S2. (**A**) Heme 8 of *S. oneidensis* MR-1 H561M MtrC with heme iron coordinating ligands Met-561 and His-580. Distances between coordinating ligand atoms and the heme iron are shown. (**B**) Same as (A) with 2Fo-Fc (blue) and Fo-Fc (green/red) electron density maps contoured at 1.5 and 3.5 sigma respectively. (**C**) Resulting 2Fo-Fc (blue) and Fo-Fc (green/red) electron density maps, contoured at 1.5 and 3.5 sigma respectively, resulting from refinement of the wild-type MtrC structure (PDB ID: 4LM8) against the H561M MtrC data. All figures display Heme 8 in cylinder representation with the iron atom represented as an orange sphere. Heme ligands are shown in ball and stick representation with oxygen atoms colored red, nitrogen atoms colored dark blue and sulfur atoms colored yellow.

Table S5. Data collection and refinement statistics for H561M MtrC (PDB ID 7O7G)

	H561M MtrC
**Data collection**	
Space group	$P\ 2_1\ 2_1\ 2_1$
Cell dimensions	
a, *b*, *c* (Å)	52.88, 89.66, 153.90
α, β, γ (°)	90.00, 90.00, 90.00
Resolution (Å)	52.90-1.60 (1.63-1.60)
R_{merge} (%)	10.2 (88.1)
$CC_{1/2}$ (%)	99.8 (67.3)
$I / \sigma I$	9.2 (0.7)
Completeness (%)	99.8 (95)
Multiplicity	5.5 (5.6)
**Refinement**	
Resolution (Å)	1.60
No. reflections	91812
R_{work} / R_{free}	0.170/0.205
No. atoms	
Protein	4787
Ligand/ion	486
Water	792
B-factors	
Protein	23.6
Ligand/ion	20.6
Water	34.9
R.m.s. deviations	
Bond lengths (Å)	0.014
Bond angles (°)	2.7

*Values in parentheses are for highest-resolution shell.

3. Analytical Ultracentrifugation

Sedimentation equilibrium (SE) experiments were performed using a Beckman Optima XLA-I analytical ultracentrifuge equipped with scanning absorbance optics. Measurements were performed with 0.4 μM protein in 50 mM Na_2HPO_4/NaH_2PO_4, 50 mM NaCl, 0.1% (v/v) Triton X-100, pH 7.5 for which the density (ρ) was calculated as 1.007 g/mL using utility software in Ultrascan II (8). This tool was also used to determine a partial specific volume ($\bar{\upsilon}$) of 0.721 mL/g from the amino acid sequence of MtrC. Sedimentation equilibrium was performed at 20 °C using speeds of 8k, 10k, and 12k rpm with absorbance profiles, Fig. S3, recorded at 410 nm. The programme Ultrascan II was used to analyze the sedimentation equilibrium profiles and the data were found to be well-described by the behavior predicted for single non-interacting species. The corresponding molecular masses, Fig. S3, are in good agreement with those from LCMS, Table S3, and indicate that the (Ru-)MtrC proteins are monomeric under the experimental conditions.

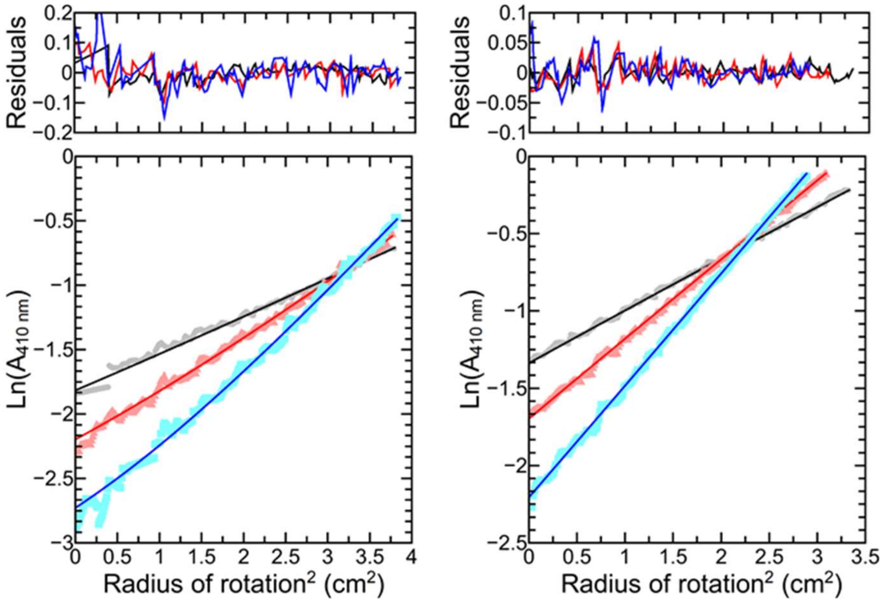

Fig. S3. Sedimentation equilibrium data for MtrC (Left) and Ru-MtrC His8 (Right). Lower panels show the natural log of absorbance at 410 nm against the square of the radius of rotation. Processed data (symbols) for one scan at each speed at 8k (gray), 10k (red) and 12k (blue) rpm and fits (lines) to the behavior for single, non-interacting species with weight average molecular masses of approximately 79.4 kDa and 82.4 kDa for MtrC and Ru-MtrC His8 respectively. Upper panels show the residuals between processed data and the fits.

4. Time-Resolved and Static Photoluminescence Spectroscopy

Anaerobic samples containing 0.7 μM of protein, Ru-dye labeled protein, or Ru(II)(4-bromomethyl-4'-methylbipyridine)$(bpy)_2(PF_6)_2$ were prepared in 20 mM TRIS-HCl, 100 mM NaCl, pH 8.5 in sealed 1 mL quartz fluorescence cuvettes. Spectra were recorded using an Edinburgh Instruments FS5 -TCSPC spectrofluorimeter with a picosecond pulsed diode laser (EPL series) at 485 nm. Data collection was for 20 min with a time window of 2 μs (500 kHz). Data analysis was performed using the Fluoracle software. To remove the fast component observed in the buffer/electrolyte and unlabeled protein samples, those datasets were subtracted from those for the Ru-labeled proteins before fitting to define decay lifetimes (τ). The best fit with the smallest number of parameters was with a bi-exponential decay, Eq. S1, with B as amplitude.

$$Intensity\ (t) = B_1 exp\left\{-\frac{t}{\tau_1}\right\} + B_2 exp\left\{-\frac{t}{\tau_2}\right\} \qquad \text{(Eq. S1)}$$

5. Optically Monitored Potentiometric Titrations

UV-visible absorbance spectra were recorded using a V-650 UV-VIS Double-Beam Spectrophotometer with temperature control (293 K) and argon flow in the sample compartment. Spectropotentiometric titrations of ≈1 μM protein in 20 mM TRIS-HCl, 100 mM NaCl, pH 8.5 were performed as described previously (9) in the presence of redox mediators. The mediators (10 μM each) were diaminodurene, phenazine methosulfate, phenazine ethosulfate, juglone (5-hydroxy 1,4 naphtoquinone), duroquinone, mendadione, anthraquinone 2,6-sulfonate and anthraquinone 2- sulfonate. Absorbance spectra (350 – 700 nm) were collected *in situ* for a range of potentials in both reductive (using sodium dithionite) and oxidative (using potassium ferricyanide) titers.

Potentiometric titration of MtrC variants monitored by electronic absorbance spectroscopy was also performed by direct protein electrochemistry using three-electrode cell configurations and an Autolab PGSTAT30 potentiostat (EcoChemie) controlled by NOVA software. Protein was adsorbed on optically transparent mesoporous nanocrystalline SnO_2 electrodes. Electrodes were prepared by covering them with ice-cold solutions of MtrC (60 μM) and the coadsorbate neomycin (50 mM) in 20 mM TRIS-HCl, 100 mM NaCl, pH 8.5. After approximately 30 min the electrode was rinsed, to remove loosely bound material, and mounted in an optical cuvette filled with anaerobic 20 mM TRIS-HCl, 100 mM NaCl, pH 8.5 and fitted with reference and counter electrodes as previously described (10). Adsorbed MtrC at ambient temperature was equilibrated at defined potentials and the electronic absorbance recorded. Spectral quality was optimized by mounting an equivalent electrode, lacking adsorbed protein, in the reference beam to minimize contributions from light scattering by the electrode. For both experiments, the change in peak absorbance at 552 nm referenced *versus* a 560 nm isosbestic point was used to monitor the extent of MtrC reduction, Fig. S4A.

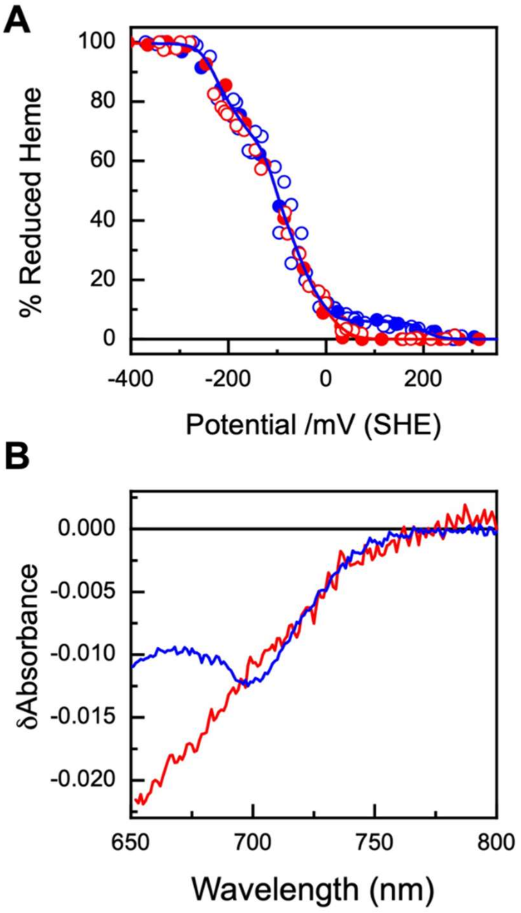

Fig. S4. Optically Monitored Potentiometric Titration of MtrC Met8 (blue), MtrC His8 (red) (A). Data are from titrations of the protein in solution (open symbols) and adsorbed states (filled symbols) with a minimum of 2 independent experiments for each (only 1 is shown for the adsorbed proteins). Line shows fits to the sum of ten contributions displaying Nernstian n=1 behavior with the E_m values of Table S6. **(B)** UV-visible absorbance difference (×20) of the spectrum of MtrC Met8 (blue) or MtrC His8 (red) after equilibrating with excess ascorbate (solution potential approx. +16 mV vs SHE (11)) *minus* the spectrum of the corresponding fully oxidized protein.

Table S6. E_m values from Potentiometric Titration of MtrC proteins.

	E_m /mV vs SHE									
MtrC Met8	-233	-223	-177	-106	-106	-106	-58	-48	-10	+199
MtrC His8	-233	-223	-177	-106	-106	-106	-57	-48	-10	+31

6. Voltammetry

Template stripped gold (TSG) working electrodes were prepared by a method described previously (12). Briefly, 150 nm gold (99.99%; Goodfellow) was evaporated on silicon wafers (IDB Technology Ltd, UK) using an Edwards Auto 306. After evaporation, 1.2 cm^2 glass slides were glued to the gold layer with Epo-Tek 377 for 2 h at 120 °C. The glass slides were detached to expose fresh TSG surfaces that were covered with a self-assembled monolayer (SAM) by incubation overnight at room temperature with a mixture of 0.8 mM 8-mercaptooctanoic acid (in water) and 0.2 mM 1-octanethiol (in ethanol). After incubation, excess thiol was gently washed away with water and the electrode was dried under a nitrogen flow.

Protein film electrochemistry was performed in a home-built electrochemical cell with a standard three-electrode setup. As the working electrode, the SAM-modified TSG was embedded in a polytetrafluoroethylene (PTFE) holder with a rubber O-ring seal, placed in a glass electrochemical cell container with a platinum wire counter electrode and a saturated silver/silver chloride reference electrode (Ag/AgCl; Radiometer analytical, France) and 2 mL of 20 mM TRIS-HCl, 100 mM NaCl, pH 8.5 was added. The working electrode surface area exposed to the buffer-electrolyte was 0.25 cm^2. Potentials are quoted *versus* SHE by addition of 0.199 V to the measured values.

The quality of the SAM was assessed with electrochemical impedance spectroscopy before the immobilization of Ru-MtrC. To form the Ru-MtrC protein film, the electrolyte was removed and the electrode exposed to 50 μL of 1 μM protein solution for 1 min at 20 °C. After rinsing the electrochemical cell more than three times with 2 mL buffer-electrolyte, making sure the electrode remains under fluid throughout, cyclic voltammograms (CVs) were obtained using an Autolab electrochemical analyzer (Ecochemie, Utrecht, Netherlands) equipped with a PGSTAT 128N potentiostat, SCANGEN and ADC750 modules, and FRA2 frequency analyzer (Ecochemie). The electrochemical cell was in a steel mesh Faraday cage to minimize electrical noise, and all experiments were conducted after purging with argon. CVs, Fig. S5, were baseline subtracted using the freely available software Q-SOAS (13).

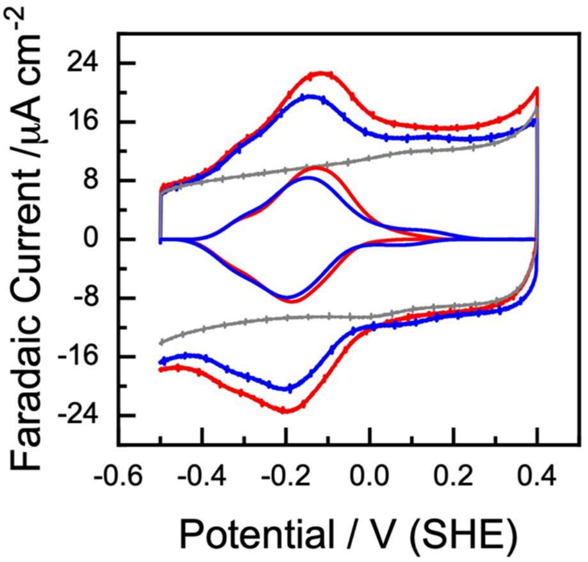

Fig. S5. Cyclic voltammograms (5 V s^{-1}) for Ru-MtrC Met8 (blue), Ru-MtrC His8 (red) and baseline (gray). Inset: baseline subtracted responses.

7. Quantification of Spectral Features in the Linear Spectra of the MtrC Proteins

To define spectral features associated solely with reduction of the His/Met ligated heme of MtrC Met8, an excess of the mild chemical reductant sodium ascorbate (11) was added to an anaerobic sample of ≈0.6 μM protein in 20 mM TRIS-HCl, 100 mM NaCl, pH 8.5. Using the extinction coefficient of MtrC Met8, derived by pyridine hemochrome (Table S4), the reduced *minus* oxidized difference spectrum was defined, Fig. S6*A* blue. This spectrum yielded the reduced *minus* oxidized difference extinction coefficients presented in Table S7 and a peak (423 nm) intensity in the Soret region of 69 mM^{-1} cm^{-1} for His/Met ligated Heme 8 (peak (423 nm) to trough (405 nm) intensity in the Soret region of ≈115 mM^{-1} cm^{-1} for a His/Met ligated MtrC heme).

Equivalent data for His/His ligated hemes was obtained by addition of sufficient of the strong reductant sodium dithionite to fully reduce hemes of Ru-MtrC His8, Fig. S6*A* red. These data produced the reduced *minus* oxidized difference extinction coefficients presented in Table S7 and a peak (421 nm) intensity in the Soret region of ≈126 mM^{-1} cm^{-1} for a His/His ligated MtrC heme (peak (421 nm) to trough (405 nm) intensity in the Soret region of ≈180 mM^{-1} cm^{-1} for a His/His ligated MtrC heme).

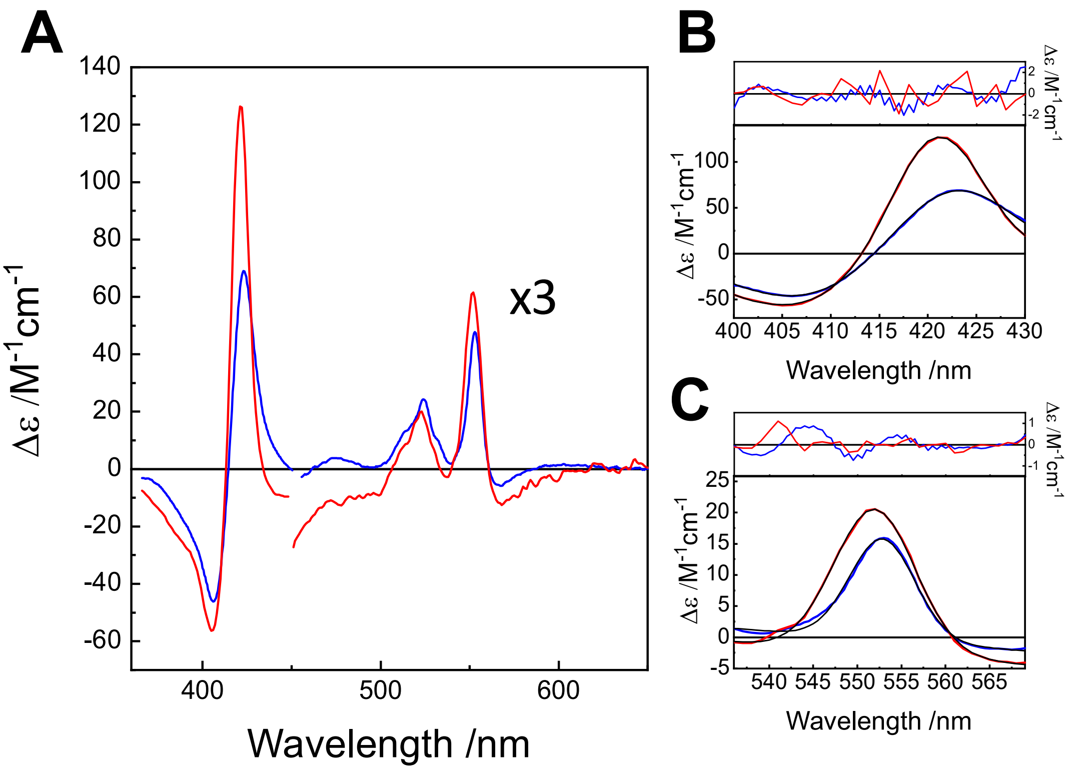

Fig. S6. Linear Reduced *Minus* Oxidized Difference Spectra for Single MtrC Hemes having His/Met (blue) and His/His (red) ligation. Difference spectra (A) with Gaussian fits (black) to the Soret (B) and Q-band (C) features. Residuals to the Gaussian fits are shown in the upper panels of (B) and (C). Samples in 20 mM TRIS-HCl, 100 mM NaCl, pH 8.5.

Table S7: Difference extinction coefficients (reduced *minus* oxidized) single His/His and His/Met ligated hemes derived from linear spectra, Fig. S6.

**Feature /nm**	**Δε /mM^{-1}cm^{-1}**
His/His Heme	
370	-10.2
421 (Soret)	126.4
475	-4.0
552 (Q-band)	20.5
His/Met Heme	
370	-3.4
423 (Soret)	69
475	1.3
553 (Q-band)	15.9

To further compare the spectral features of His/His *versus* His/Met *c*-type hemes in these proteins, a lineshape fit was performed. In the spectral range of 400 to 430 nm (Soret region) the sum of two Gaussian lineshapes was used (Eq. S2) to fit the negative contributions from Fe^{3+} heme and the positive Fe^{2+} heme features (Fig. S6*B*),

$$G(\lambda) = \frac{A_1}{w_1\sqrt{\frac{\pi}{4\ln}}} e^{\left(\frac{-4ln2(\lambda-\lambda c_1)^2}{w_1^2}\right)} + \frac{A_2}{w_2\sqrt{\frac{\pi}{4\ln 2}}} e^{\left(\frac{-4ln2(\lambda-\lambda c_2)^2}{w_2^2}\right)} \qquad \text{(Eq. S2)}$$

where A_i is area, λc_i the central wavelength and w_i the width for the Fe^{2+} (i =1) and Fe^{3+} (i = 2) features.
NB/ This wavelength region was chosen for direct comparison to the fitting of the transient absorbance data which is limited by the noise from excitation pulse at 457 nm (Section 8.2, Fig S9).

In the spectral region 535 to 569 nm that includes the Q-band, a single Gaussian lineshape was used (Eq. S3) to define the sharp positive features from Fe^{2+} heme (Fig. S6*C*). In this spectral region features from Fe^{3+} are fairly featureless and are accounted for by the slope b and offset y_0 applied.

$$G(\lambda) = \frac{A}{w\sqrt{\frac{\pi}{4\ln}}} e^{\left(\frac{-4ln2(\lambda-\lambda c)^2}{w^2}\right)} + y_0 + b\lambda \qquad \text{(Eq. S3)}$$

The area, A, is an extinction coefficient (per area) for each single heme species (His/His or His/Met *c*-type heme) and is clearly larger for a His/His heme in comparison to a His/Met heme in both the Soret and Q-band regions (see Table S8 – linear difference spectra lineshape fitting coefficients).

It is clear from Fig. S6*B* (and Fig. 2*B*, main text) that the bandwidth, w, of the Soret Fe^{2+} peak is substantially wider in a His/Met heme (≈14 nm) in comparison to a His/His heme (≈11 nm) and this feature is used to calculate the percentage of each species in the TAS data shown later. There are also slight variations in the central wavelengths λc of the heme types in the linear difference spectra.

Table S8: Lineshape fitting coefficients of linear difference spectra of single His/His or His/Met *c*-type heme derived from data of Fig. S6.

Feature	λ_c /nm	w /nm	A /mM^{-1} cm^{-1}
His/His heme			
Soret Fe^{2+}	421.0	10.9	1553.6
Soret Fe^{3+}	405.2	18.0	-1078.1
Q-band Fe^{2+}	552.0	10.8	265.4
His/Met heme			
Soret Fe^{2+}	422.7	13.9	1084.1
Soret Fe^{3+}	406.5	18.0	-909.4
Q-band Fe^{2+}	552.8	8.7	150.2

8. Transient Absorbance Spectroscopy (TAS)

TAS was performed using the Time-Resolved Multiple-Probe Spectroscopy (TRMPS) facility at the Central Laser Facility of the Rutherford Appleton Laboratory, as previously described (14). Two separate sets of experiments were performed to probe the spectral changes in the regions of 350 – 440 nm and 470 – 650 nm using Ru-labeled proteins at two concentrations: 5 μM and ≈150 μM respectively. In each set of experiments an appropriate cut-off optical filter was used to block the scattered 457 nm excitation light (short-pass filter when probing 350-440 nm region and long-pass filter when probing 470-650 nm region).

Measurements were performed in pairs, first Ru-MtrC and then the corresponding unlabeled protein (MtrC His8 or Met8) at a similar concentration, with the experimental conditions as close as possible for both measurements. A baseline spectrum at -500 fs (before pump pulse) was subtracted from all transient spectra. For Ru-MtrC, in order to extract spectra describing consequences of excitation of only Ru dye, and not direct into the heme, the direct heme excitation of Y657C MtrC was subtracted as described previously (14) for equivalent experiments with a tetra-heme cytochrome labeled with the Ru dye used in this study. Direct heme excitation (by excitation at 457 nm) has fully decayed by 100 ps which, for Ru-MtrC, is considerably faster than the lifetimes extracted for electron transfer from Ru to the MtrC protein.

8.1 Analysis of TAS Spectral Contributions

Scheme 1 (main text) shows the different species in the Ru-MtrC photocycle, namely the Ru-MtrC ground state (GS), the ^{3}Ru-MtrC excited state (ES) and the Ru^+-$MtrC^-$ charge-separated state. All three species have overlapping spectral features as described by Fig. S7.

On excitation, at 457 nm, of $Ru(bpy)_3$ without protein attached, there are overlapping TAS features (Fig. S7, green line) that are well described by the literature (15-17) as the ground state bleach and excited state absorbance. These spectral features have a lifetime of 462 ns in agreement with the value measured by time-resolved photoluminescence, Fig. 2*C*, main text.

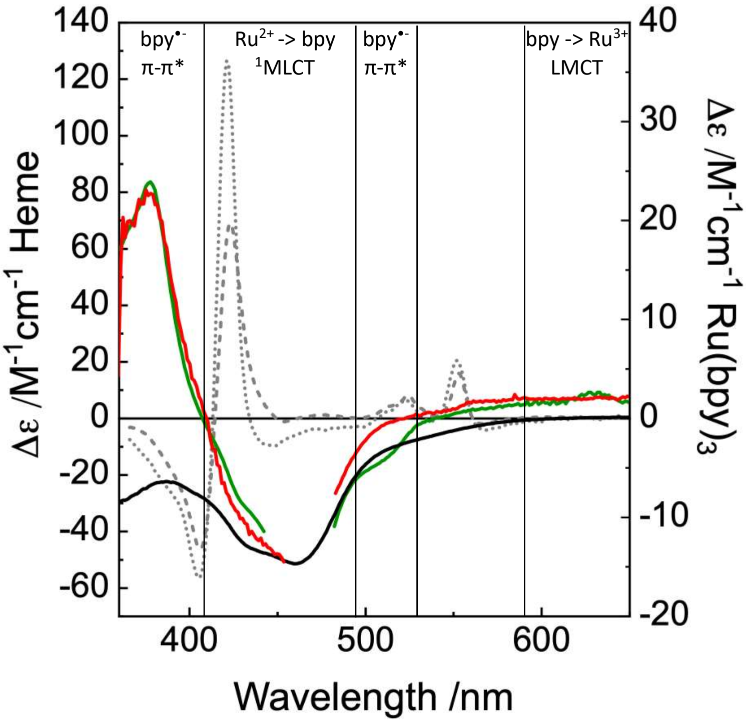

Fig. S7. Deconvolution of the spectral TAS features. The solid lines are the spectral features attributed to the $Ru(bpy)_3$ without protein attached (green); Ru-MtrC His8 at early times after excitation (5 ps) (red). The inverted ground state linear absorbance spectrum of $Ru(bpy)_3$ without protein attached is shown (black). As a comparison, the linear difference absorbance spectra of the His/His (dotted gray) and His/Met (dashed gray) *c*-type hemes (from Fig. S6*A***)** are shown.

TAS of Ru-MtrC His8 (Fig. S7, red line) at early times after excitation (<10 ps) reveals features similar to those from $Ru(bpy)_3$ alone as charge injection is yet to occur. Those features include a trough (bleach) at ≈450 nm which is predominantly attributed to the loss of the ground state (GS) $Ru(bpy)_3$ ^{1}MLCT transition (15-17). This feature can be used directly to measure the concentration of the Ru-MtrC making the following assumptions:

1) the excited state (ES) absorption of the Ru-dye (^{3}Ru-MtrC) does not contribute to the bleach signal at ≈450 nm. When $Ru(bpy)_3$ is attached to TiO_2, it has been shown that there is no ES relaxation at 450 nm following charge injection into the TiO_2 (15, 16);
2) when the ES has fully decayed (after charge injection into a protein or TiO_2), the negative GS feature is directly comparable to the inverse GS absorption spectrum of $Ru(bpy)_3$ (Fig. S7, black line) as well as the electrochemically-derived (Ru^{3+} *minus* Ru^{2+}) difference spectrum (16) and all have an extinction coefficient at 452 nm of -14.6 mM^{-1} cm^{-1} (18), Table S9.

Note: the extinction coefficient for $Ru(bpy)_3$ is substantially lower than that of the heme Soret band.

The TAS peak centered at 370 nm is predominantly an ES feature attributed to a $bpy^{\bullet-}$ π - π* transition of ^{3}Ru-MtrC (19). There is, however, a small bleach contribution from the GS $Ru(bpy)_3$ that must be subtracted to determine the ES ^{3}Ru-MtrC concentration correctly. The extinction coefficient for the positive feature at 370 nm can be determined from the measured Ru-MtrC TAS data before charge injection occurs (<10 ps) by comparison of the 370 nm absorbance to the trough (bleach) at 452 nm with known extinction coefficient of 14.6 mM^{-1} cm^{-1} (18), Table S9. The GS $Ru(bpy)_3$ contribution was determined using the inverse $Ru(bpy)_3$ linear absorption spectrum at 370 nm (15, 16) when normalized to the extinction coefficient of the trough (bleach) at 452 nm and subtracted to give an extinction coefficient for the ES ^{3}Ru-MtrC only. An assumption was made that the oxidized $Ru(bpy)_3^{3+}$ does not contribute at 370 nm.

The TAS bands >500 nm are assigned to a secondary ES bpy$^{\bullet-}$ π - π^* transition (500-530 nm) (20) and an ES ligand to metal charge transfer transition from neutral bpy to Ru^{3+} (575 - 665 nm) (15, 20). We note that the secondary bpy$^{\bullet-}$ π - π^* transition is only seen for the Ru-MtrC (Fig. S7, red line).

8.2 Time-Dependent Concentration of Ru^+-$MtrC^-$ (Fe^{2+})

Due to large differences in intensity between the Soret and Q-band peaks of the heme spectrum, two different protein concentrations were used to keep a good signal-to-noise across the full wavelength region: 5 μM (350 – 440 nm region) and ~150 μM (470 – 650 nm region), respectively. The resulting TAS data (Fig. 3*A*, main text) was normalized to the ^{3}Ru:MtrC peak at 370 nm at 5 ps (before charge injection occurs). The data in the 470 – 650 nm region was divided by a Normalization Factor (Equation S8) so that the [Fe^{2+}] concentration derived from lineshape analysis of the Q-band data matched that from the Soret data. . The Normalization Factors were 6.9 and 5.7 for Ru-MtrC Met8 and Ru-MtrC His8 data, respectively. Fig. S8 shows the TAS data for the Fe^{2+} of Ru-MtrC Met8 (upper panel) and Ru-MtrC His8 (lower panel) after the Normalization Factor has been applied. All subsequent values used for the calculations of Ru-MtrC and ^{3}Ru-MtrC were taken from the normalized data.

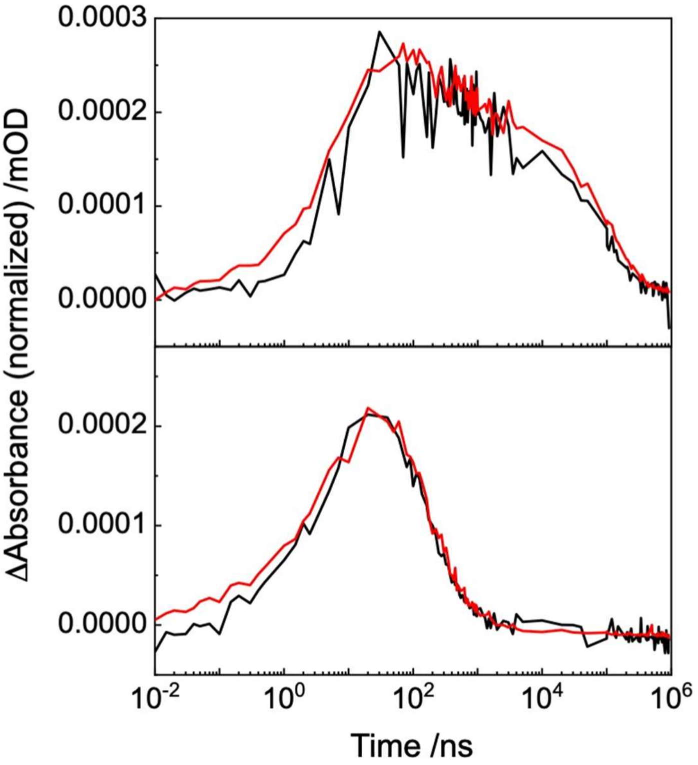

Fig. S8. Fe^{2+} TAS intensity of 5 μM (black) versus 150 μM (red) samples for Ru-MtrC Met8 (upper panel) and Ru-MtrC His8 (lower panel). The 150 μM dataset is normalized to the 5 μM dataset by a Normalization Factor of 6.9 and 5.7 for Ru-MtrC Met8 and Ru-MtrC His8, respectively (see Equation S8).

In the Soret spectral range of 400 to 430 nm (Fig. S9*A*, Fig. 3*A* main text) the features at each time delay Δt are described by the sum of two Gaussian lineshapes on a sloping offset to account for the $Ru^{2+}(bpy)_3$ contribution (14). A negative Gaussian lineshape accounts for the trough describing loss of Fe^{3+} heme. A positive Gaussian lineshape accounts for the peak describing gain of Fe^{2+} heme. The corresponding equation is Eq. S4:

$$G(\lambda, \Delta t) = \frac{A_1(\Delta t)}{w_1\sqrt{\frac{\pi}{4\ln 2}}} e^{\left(\frac{-4ln2(\lambda-\lambda c_1)^2}{w_1^2}\right)} + \frac{A_2(\Delta t)}{w_2\sqrt{\frac{\pi}{4\ln 2}}} e^{\left(\frac{-4ln2(\lambda-\lambda c_2)^2}{w_2^2}\right)} + y_0 + b(\Delta t)\lambda \qquad \text{(Eq. S4)}$$

with time-dependent parameters area A_1, A_2, central wavelength λc_1, λc_2, and bandwidth w_1, w_2, for the Fe^{2+}, Fe^{3+} peaks, respectively, offset y_0, and slope b.

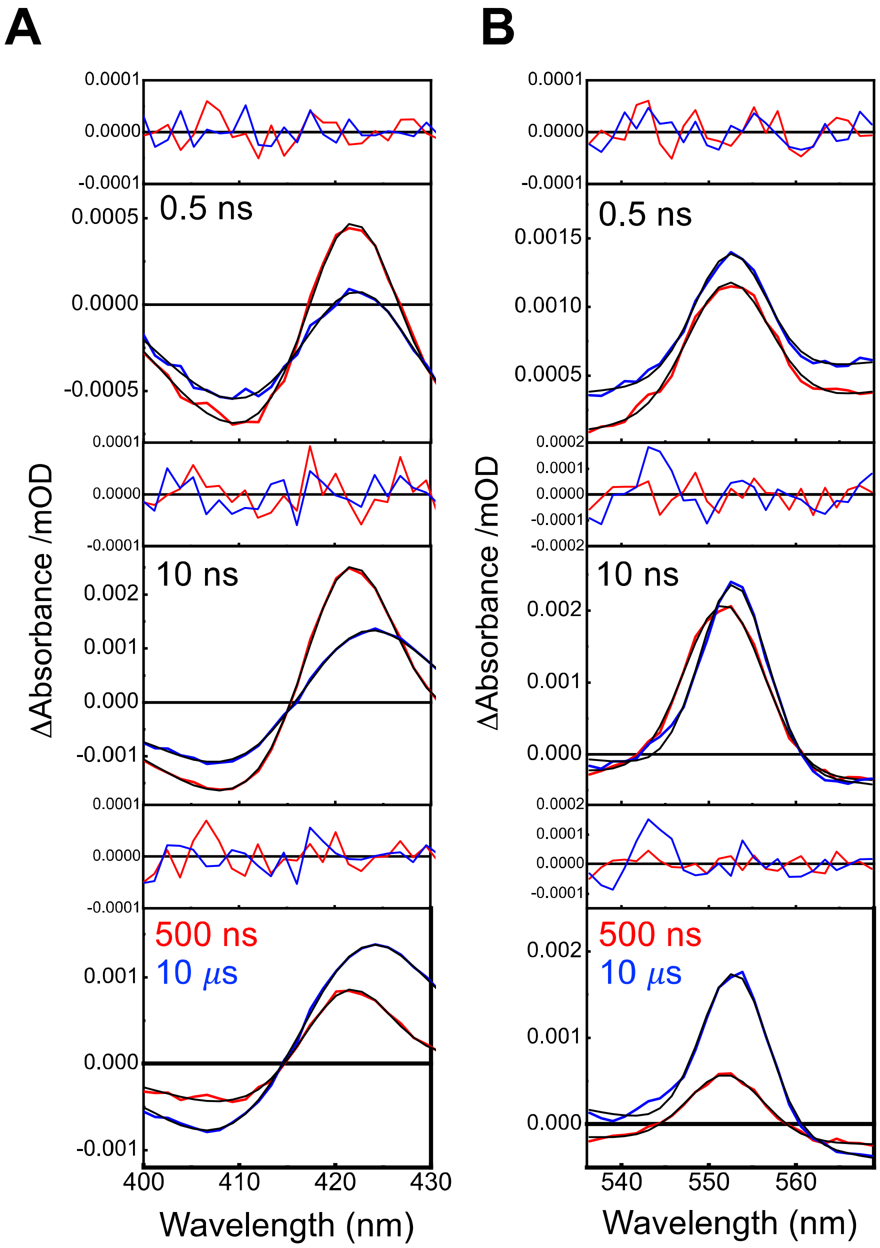

Fig. S9. Transient Absorbance for (A) the Soret band and (B) the Q-band. Data for Ru-MtrC Met8 (blue) and Ru-MtrC His8 (red) at the indicated time points. Fits (black) as described in the text. Residuals (upper panels). Measurements in 20 mM TRIS-HCl, 100 mM NaCl, pH 8.5.

In the Q-band spectral range of 535 to 569 nm (Fig. S9*B*) the features at each time delay Δt are described by a single Gaussian lineshape describing absorbance by the Fe(II) heme superimposed on an offset (y_0) and slope (b) to account for the negative Fe^{3+} bleach as well as any background $Ru^{2+}(bpy)_3$ contribution (14). The corresponding equation, Eq. S5, has time-dependent parameters area A, central wavelength λc, and bandwidth w for the Fe^{2+} peak at 552 nm.

$$G(\lambda, \Delta t) = \frac{A(\Delta t)}{w\sqrt{\frac{\pi}{4\ln 2}}} e\left(\frac{-4ln2(\lambda - \lambda c)^2}{w^2}\right) + y_0 + b(\Delta t)\lambda \qquad \text{(Eq. S5)}$$

Initial values for w and λ_c, used in fitting the protein spectral data were those of the linear difference spectral fits (Table S8). For both spectral regions, the fitting areas A, were converted into concentrations of Fe^{3+} (Fe^{2+}) heme by direct comparison to the corresponding parameters derived from Gaussian fits to the linear difference spectra (Fig. S6, Table S8). The validity of the fitting coefficients was confirmed when the concentration of Fe^{3+} lost and concentration of the Fe^{2+} formed, as derived from the lineshape analysis, were compared and found to be equal.

Table S9. Extinction coefficients for Ru-MtrC and ^{3}Ru-MtrC at 475 and 370 nm, respectively, derived from the inverted absorbance of $Ru(bpy)_3$ and the TAS of Ru-MtrC at early time, before charge injection.

**Wavelength /nm**	
**$Ru^{2+}(bpy)_3$ (inverted linear absorbance)**	**ε /mM^{-1}cm^{-1}**
452	-14.6 (18)
370	-6.6 *(derived from spectrum)*
**Ru-MtrC TAS**	**$\Delta\varepsilon$ /mM^{-1}cm^{-1}**
452	- 14.6 (18)
370	22.5 *(derived from TAS at 5 ps)*
475	-7.0 *(derived from TAS at 5 ps)*
**^{3}Ru-MtrC (bpy$^{\bullet -}$, π - π*)**	**ε /mM^{-1}cm^{-1}**
370	29.1 *(derived from above values)*

For Ru-MtrC Met8 the percentage of Fe(II) hemes having His/Met (His/His) ligation at each time delay was calculated from the bandwidth, w, of the positive contribution to the Soret feature. This feature is substantially wider for a His/Met heme (14 nm) when compared to a His/His heme (11 nm), Fig. 3*B* main text. The total difference in bandwidth, δw, between a fully His/Met heme and a fully His/His heme is therefore 3 nm. For each time delay, Δt, the difference in bandwidth compared to fully His/His heme (11 nm) was calculated and divided by the maximum difference of 3 nm and converted to a % His/Met heme. To minimize the noise, a rolling average of 6 points was used. The % His/Met (His/His) heme was used to calculate the concentration of each heme type.

8.3 Time-Dependent Concentrations of Ru-MtrC and ^{3}Ru-MtrC

Following the above assumptions, time-dependent concentrations of Ru-MtrC and ^{3}Ru-MtrC can be calculated from the TAS data using wavelengths of 475 nm and 370 nm, respectively, and the derived extinction coefficients shown in Table S9. The 475 nm wavelength was chosen as it was away from both the excitation pulse (457 nm) and the secondary ES bpy$^{\bullet -}$ π - π* transition.

As the protein heme features also overlap with the Ru(bpy)3 GS and ES features, these need to be subtracted before the time-dependent concentrations of Ru-MtrC and ^{3}Ru-MtrC are calculated:

$$[\mathrm{Ru-MtrC}]\,(\Delta t) = \frac{CF \times \Delta OD_{475nm}(\Delta t) - (CF \times [Qband\ Fe^{2+}](\Delta t) \times \Delta\varepsilon_{475nm}^{MtrC} \times l)}{l \times \Delta\varepsilon_{475nm}^{Ru-Mt\quad\_TAS(t,5ps)}} \qquad \text{(Eq. S6)}$$

$$[\,^{*}\mathrm{Ru-MtrC}]\,(\Delta t) = \frac{\Delta OD_{370nm}(\Delta t) - (CF \times [Qband\ Fe^{2+}](\Delta t) \times \Delta\varepsilon_{370nm}^{MtrC} \times l) - ([\mathrm{Ru{-}MtrC}](\Delta t) \times \varepsilon_{370nm}^{Ru(bpy)3} \times l)}{l \times \varepsilon_{370nm}^{*Ru-MtrC}} \qquad \text{(Eq. S7)}$$

where [Ru-MtrC] (Δt) and [^{3}Ru-MtrC] are the concentrations of Ru-MtrC and ^{3}Ru-MtrC to be determined, ΔOD_{475nm} (Δt) and ΔOD_{370nm} (Δt) are the single point values from the TAS spectra at 475 nm and 370 nm respectively, the Normalization Factor, NF, is

$$NF = \frac{[Soret\ FeII](\Delta t)}{[Qband\ FeII](\Delta t)} \qquad \text{(Eq. S8)}$$

[Qband FeII] (Δt) is the concentration of Fe^{2+} determined from the TAS lineshape fits of the Q-band data (535 to 569 nm), $\Delta\varepsilon_{475nm}^{MtrC}$ and $\Delta\varepsilon_{370nm}^{MtrC}$ are the extinction coefficients from the linear (His/His or His/Met) heme difference spectra at 475 nm and 370 nm (Table S8) , l is the pathlength, $\Delta\varepsilon_{475nm}^{Ru-MtrC\_TAS(t,5ps)}$ and $\Delta\varepsilon_{370nm}^{Ru-MtrC\_TAS(t,5ps)}$ are the extinction coefficients obtained from the Ru-MtrC TAS data at 475 nm and 370 nm at 5 ps (Fig. S7 red line, Table S9), $\Delta\varepsilon_{370nm}^{Ru(bpy)3}$ is the extinction coefficient from the inverted linear Ru^{2+}(bpy)3 spectrum at 370 nm (Fig. S7 black line, Table S9) and

$$\varepsilon_{370nm}^{*Ru-MtrC} = \Delta\varepsilon_{370nm}^{Ru-Mt\quad\_TAS(t,5ps)} - \varepsilon_{370nm}^{Ru(bpy)3\ GS} \qquad \text{(Eq. S9)}$$

The calculated time-dependent concentrations for Ru^{+}-$MtrC^{-}$ (Fe^{2+}), Ru-MtrC and ^{3}Ru-MtrC were converted to populations by a normalization factor so that ^{3}Ru-MtrC =1 and Ru-MtrC = ^{3}Ru-MtrC at time = 1 – 5 ps, before charge-separation occurs.

9. Fitting the transient populations

We describe the concentration decay of ^{3}Ru-MtrC by the following rate equation:

$$\mathrm{d}(p(\,^3\mathrm{Ru{-}MtrC_x}))/\mathrm{d}t = -k_{CS}^{x} \cdot p(\,^3\mathrm{Ru-MtrC_x}) \qquad \text{(Eq. S10)}$$

where k_{CS}^{x} is the rate constant for charge separation for conformer *x* of the Ru-label, *x* = a, b, c,…

The data for ^{3}Ru-MtrC His8 and ^{3}Ru-MtrC Met8 are very similar but the data for the latter exhibit relatively high noise for times > 50 ns, which prevents a good fit of the concentration decay at long times. Hence we decided to fit the combined data of ^{3}Ru-MtrC His8 and ^{3}Ru-MtrC Met8 to a single set of rate constants k_{CS}^{x} and % contribution of conformer *x*. This is justified also by the very similar charge separation rates and % contributions for the two proteins from photoluminescence spectroscopy, Table 1 main text. We investigated 4 models comprised of one (x = a), two (x = a, b), three (x = a, b, c) and four (x = a, b, c, d) kinetically distinct conformations of the Ru-label. For each case, more than 5000 initial sets of fit parameters were scanned, and each set was refined to minimize (locally) the weighted sum of squares of the residuals. The set of fit parameters resulting in the smallest residuals is taken. The results are plotted in Fig. S10. One can clearly see that 3 conformers are required to fit the data well (R^2 =0.991), whereas more than 3 conformers do not further improve the fit any further (R^2 =0.992). The k_{CS}^{x} and % contributions for the final 3-conformer model are

summarized in Table 2 main text.

After fixing the k_{CS}^{x} and % contributions, we fit the concentrations of Ru^{+}-$MtrC^{-}$ for the His8 and Met8 proteins separately to the following set of rate equations describing Scheme 1 main text

$$\mathrm{d}(p(CS_x^{10}))/\mathrm{d}t = k_{CS}^{x} \cdot p(\,^3\mathrm{Ru} - \mathrm{MtrC_x}) + k_{10,9} \cdot p(CS_x^{9}) - (k_{9,10} + k_{CS}^{x}) \cdot p(CS_x^{10}) \quad \text{(Eq. S11)}$$
$$\mathrm{d}(p(CS_x^{9}))/\mathrm{d}t = k_{9,10} \cdot p(CS_x^{10}) + k_{9,8} \cdot p(CS_x^{8}) - (k_{10,9} + k_{89}) \cdot p(CS_x^{9}) \quad \text{(Eq. S12)}$$
$$\mathrm{d}(p(CS_x^{8}))/\mathrm{d}t = k_{8,9} \cdot p(CS_x^{9}) - k_{9,8} \cdot p(CS_x^{8}) \quad \text{(Eq. S13)}$$
$$\mathrm{d}(p(\mathrm{Ru{-}MtrC_x}))/\mathrm{d}t = k_{CR}^{x} \cdot p(CS_x^{10}), \quad \text{(Eq. S14)}$$

to obtain the rate constants $k_{10,9}$, $k_{9,10}$, $k_{8,9}$, $k_{9,8}$ and k_{CR}^{x}. More than 5000 initial sets of fit parameters were scanned for each fit, and each set was refined to minimize (locally) the weighted sum of squares of the residuals. The set of fit parameters resulting in the smallest residuals is taken for the Ru-MtrC His8 fitting. For the fitting of Ru-Met Met8, the set of fit parameters giving the smallest residuals resulted in an unphysically big k_{CR} rate constant – this set was discarded. The set of fitting parameters giving the second smallest residuals was chosen instead. The chosen fits to the experimental TAS data are shown in Fig. 4 main text, R^2 values of these fits are summarized in Table S10 and the corresponding rate constants are summarized in Tables 2 and 3 main text.

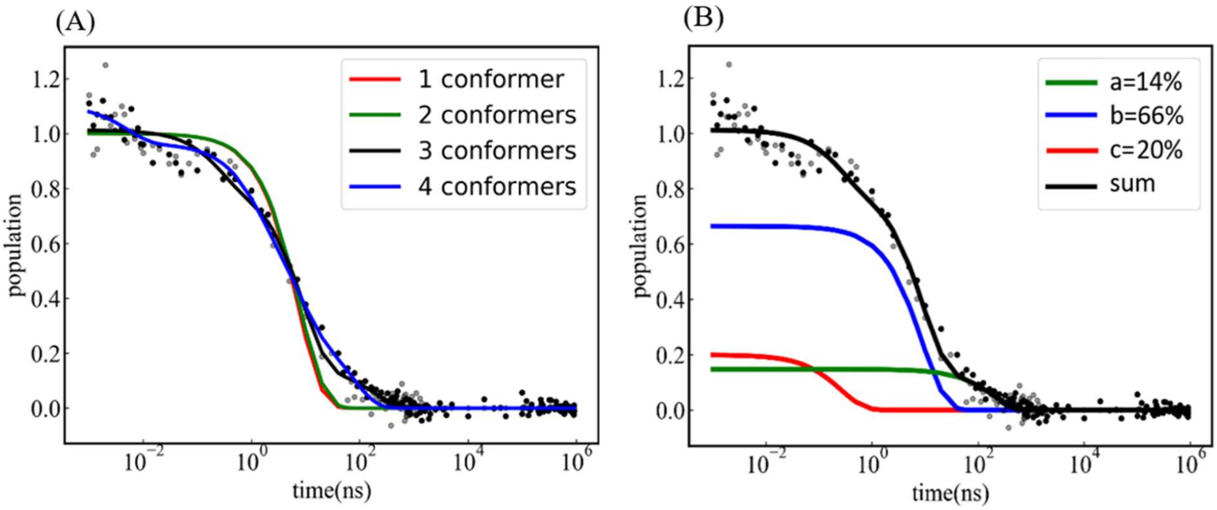

Fig. S10. Fit of combined TA data for ^{3}Ru-MtrC His8 (black scatter points) and ^{3}Ru-MtrC Met8 (grey scatter points). (A) Fit for different number of conformers. (B) Fit of the 3-conformer model (black line) broken down in contributions from conformers a, b and c with k_{CS}^{x} and % contributions in Table 2 main text.

Table S10. Summary of R^2 values of fits to the TAS data shown in Fig. 4 main text.

protein	^{3}Ru-MtrC	Ru^{+}-$MtrC^{-}$	Ru-MtrC
Ru-MtrC His8	0.991	0.965	0.991
Ru-MtrC Met8	0.991	0.966	0.891

10. Computed rate constants $k_{8,9}$ and $k_{9,8}$ for Ru-MtrC Met8

The rate constants for electron transfer between His/His Heme 9 and His/Met Heme 8 of the Ru-MtrC Met8 protein, $k_{8,9}$ and $k_{9,8}$, are obtained from the non-adiabatic (Marcus) rate equation using calculated values for electronic coupling, reorganization free energy and driving force.

Electronic coupling between the two heme cofactors was obtained as follows. An equilibrated MD snapshot from a trajectory of the wild type MtrC was taken from Ref (21), where Heme 8 and Heme 9 were half reduced. Residue 561 was changed from histidine to methionine and the resultant mutant (MtrC Met8 using the terminology of the current paper) equilibrated for 60 ns. An ensemble of approximate transition state structures for ET between heme pair 8-9 of MtrC Met8 was generated by running MD simulations for 50 ns where heme 8 and 9 were in the half-reduced state. From this trajectory 25 equidistantly spaced snapshots were extracted using the QM model termed “final model” in Ref. (21). Electronic coupling calculations were carried out with the projector-operator diabatization (POD) approach (22) on these configurations for the doublet ground state. For each configuration, the POD calculation was carried out at PBE level and the coupling between HOMO orbital of the donor, His/His Heme 9, and the LUMO orbital of the acceptor, His-Met Heme 8 was extracted. The coupling values were scaled by a factor of 1.394, which brings POD/PBE couplings in excellent agreement with high-level ab-initio data for the HAB11 database of electronic couplings (mean relative error 8.9%) (22). The final coupling value between heme pair 8-9 of MtrC Met8, 6.3 meV, was obtained by averaging the square couplings over the 25 configurations.

Reorganization free energy was obtained as a sum of inner and outer-sphere contributions. The inner-sphere contributions of His/Met Heme 8, modelled as Fe-porphyrin axially ligated by dimethyl sulfide and methyl-imidazole was 25 meV at PBE level (23), and the contribution of bis-His Heme 9, modelled as Fe-porphyrin axially ligated by two methyl-imidazoles was also 25 meV at PBE level (24), giving a total inner-sphere contribution of 50 meV. The outer-sphere contribution was taken to be the same as for Ru-MtrC His8, 0.68 eV, giving a total reorganization energy of 0.73 eV.

The driving force for Heme 9 to Heme 8 ET in Ru-MtrC Met8 was obtained using a combination of computed and experimental heme reduction potentials. At first, 10 heme reduction potentials of native MtrC were obtained by fitting the experimental potentiometric titration in terms of single heme contributions, see Fig. 2A main text, Fig. S4 and Table S6. Then the 10 computed heme microscopic reduction potentials for native MtrC were taken from Ref. (21) and shifted uniformly so as to minimize the residual error with respect to the experimental potentials. This procedure allowed one to assign the 10 experimental reduction potentials to the 10 hemes in native MtrC. In particular, we obtain an experimental potential for Heme 8 His of -0.223 V, compared to -0.236 V from computation. Potentiometric titration of the MtrC Met8 protein gave one distinct high potential peak with E_m = 0.199 V, which is assigned to the His-Met Heme 8, see Fig. 2A main text and Fig. S4. Hence, the experimental redox potential shift due to His→Met mutation is 0.199-(-0.223) = 0.422 V. This shift is added to the computed redox potential of Heme 8 in the native MtrC protein to obtain a ‘computed’ estimate for the reduction potential of Heme 8 in the MtrC Met8 protein, -0.236 V + 0.422 V = 0.186 V. The computed reduction potential of Heme 9 in MtrC Met8 is assumed to be the same as in native MtrC, -0.114 V. Therefore the computed driving force for Heme 9 to Heme 8 ET in MtrC Met8 is -0.114-0.186 = -0.30 eV.

Table S11. Computed microscopic reduction potentials of Hemes 8 and 9 in MtrC His8 and MtrC Met8.

Heme	MtrC His8 (V)	MtrC Met8 (V)
8	-0.236	0.186
9	-0.114	-0.114

11. Docking and molecular dynamics simulations of Ru-MtrC

11.1 Equilibration simulation of wild-type MtrC

The structure of the wild-type MtrC protein (PDB ID: 4LM8 (4)) was prepared in the all-oxidized state with all protein residues in the standard protonation states at pH = 7. The protein was solvated in a water box of 38060 water molecules with 28 Na^+ counterions added to neutralize the system. The system was initially minimized for 5000 steps and subsequently equilibrated for 100 ps with all protein atoms kept frozen. The temperature was rescaled to 300 K every 5000 steps and Langevin barostat was applied with a target pressure of 1.013 bar. The protein was then slowly released by applying harmonic restraints around the crystallographic positions with force constants of 99, 25, 1.0, 0.1, and 0.001 kcal/mol/A^2. The duration of each of these runs was 100 ps, the MD time step was 1 fs, the temperature was rescaled as before and the volume was held constant. Eventually, all position restraints were dropped and the protein was equilibrated for 10 ns in NPT ensemble and then 10 ns in NVT ensemble using a time step of 2 fs, Langevin thermostat and barostat with target temperature and pressure of 300 K and 1.013 bar, respectively.

11.2 Structural models for Ru-Y657C

Starting from the last snapshot of the equilibrated wild-type MtrC trajectory, all water molecules and the two closest sodium counterions were removed. The residue 657 (Tyr 657) was replaced by a cysteine and the hydrogen atom of S-H is replaced by the Ru(bpy)$_2$(4-methylbipyridene-4'-CH_2-) label (created with GaussView (25)) such that the S-C bond is 1.8 Å and the C-S-C bond angle is 98°, in accord with standard force field parameters. The orientation of the Ru-label with respect to the protein is determined by 3 dihedral angles, (τ_1, τ_2, τ_3). For detailed definition of the three dihedral angles please refer to our previous study (14). We generated a large number of docking structures in the conformational space spanned by the three dihedral angles to obtain the most stable conformations of the ligand relative to the protein as initial structures for the MD simulation. To this end, we sampled the three dihedral angles between 0° to 360° in increments of 5° to generated more than 100,000 trial structures. The total energy of each trial structure was evaluated with implicit solvation at 0.1 M and with the positions of the 26 counterions fixed. The energies were plotted against the three dihedral angles. There were 3400 structures that were within 20 kcal/mol of the lowest-energy structure, and these structures were then energy minimized with protein and counterions fixed for 100 steps and clustered with respect to the three dihedral angles. This resulted in a total of five unique low-energy clusters termed in the following conformers that are described by the following sets of dihedrals, (110°, 137°, 83°), (123°, 96°, -81°), (154°, -67°, 105°), (118°, 75°, -109°), and (161°, -107°, 75°), as shown in Fig. S11. The five conformers are used as initial structures for molecular dynamics (MD) simulations.

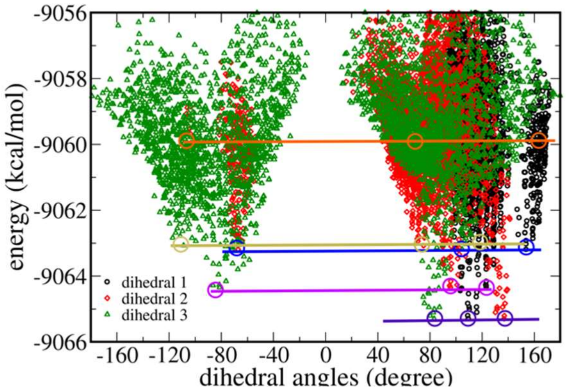

Fig. S11. Energy of docking structures after local optimization. Each docking structure is represented by three data points placed at the same energy and at the respective values for the dihedral angles, dihedral 1 (black), dihedral 2 (red), dihedral 3 (green). Five unique clusters in the space of the three dihedrals were identified as indicated by a horizontal line, and termed as conformers in the text.

11.3 Molecular dynamics for Ru-Y657C

The AMBER03 force field was used for docking and MD simulation together with the TIP3P water model and the monovalent ion parameters for Na^+ and Cl^-. The force field parameters for the heme cofactors, the axial histidine ligand, and the Ru(bpy)$_2$(4-methylbipyridene-4'-CH$_2$-) label were taken from our previous work (14).

For MD stimulations, 84 Na^+ and 58 Cl^- counter ions were added corresponding to an ionic strength of 0.1 M. The five conformers from the molecular docking results were solvated with a shell of 15 angstroms yielding a total of 39514, 39515, 39514, 39514, 39517 water molecules, respectively. They were used to initialize 5 separate MD simulations. For each simulation, the systems were equilibrated with 10 ns in NPT ensemble followed 10 ns in NVT ensemble with Langevin thermostat targeted at 300 K and Langevin barostat targeted at 1.013 bar. After that, 40 ns production run of each system was carried out at NVT ensemble, with the same setting as before. All hemes were in the oxidized state and the Ru-label was in the reduced state. Simulation timestep of 2 fs was used. All minimization and MD simulation were performed with NAMD code (26).

In Fig. S12 the 5 trajectories are shown color-coded indicating the instantaneous conformation of the Ru-label (note t=0 corresponds to the start of the production run). During the 5 × 40=200 ns simulation, we observed altogether 8 different conformers, **1-8,** with Ru-label Heme 10 edge-to-edge distances spanning 5.3-8.4 Angstroms. Two conformers (conformers **1** and **2**, see Fig. S13, black and red conformers) were observed in all 5 trajectories. They appeared to be stable on the 10 ns time scale and exhibited the smallest distances, 5.3 and 5.6 Angstroms, respectively. The rest of the conformers (conformers **3** - **8**) appeared to be transient, and they were further away from Heme 10 (see Fig. S13, cyan conformer). Edge-to-edge distances between the Ru-label and Heme 9 (Heme 8) are on average 9 (13) Angstroms longer than for Heme 10 (average over trajectory 1). This excludes direct electron injection to Hemes 9 and 8, which would bypass Heme 10.

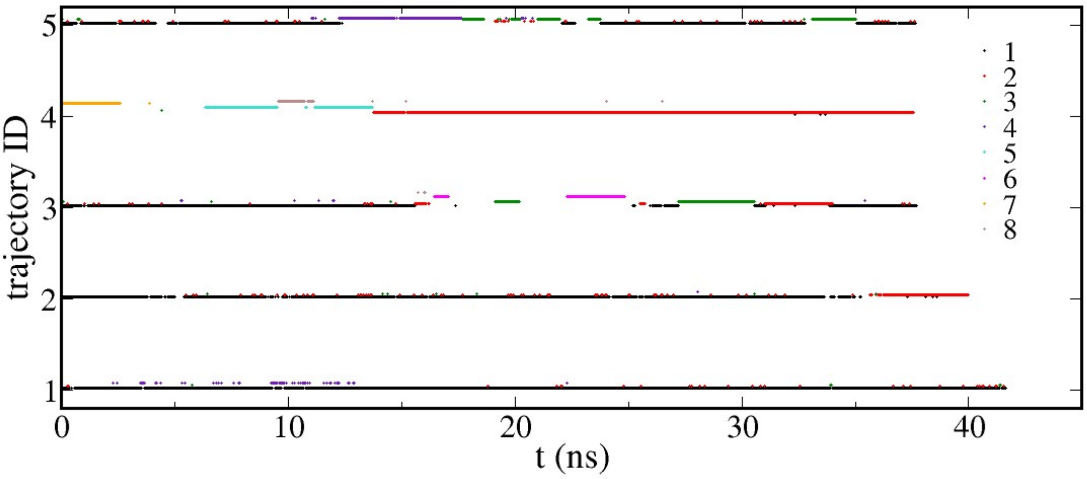

Fig. S12 Five MD simulations of Ru-Y657C starting from the five low-energy docking structures. The instantaneous conformation of the Ru-label is color-coded as indicated.

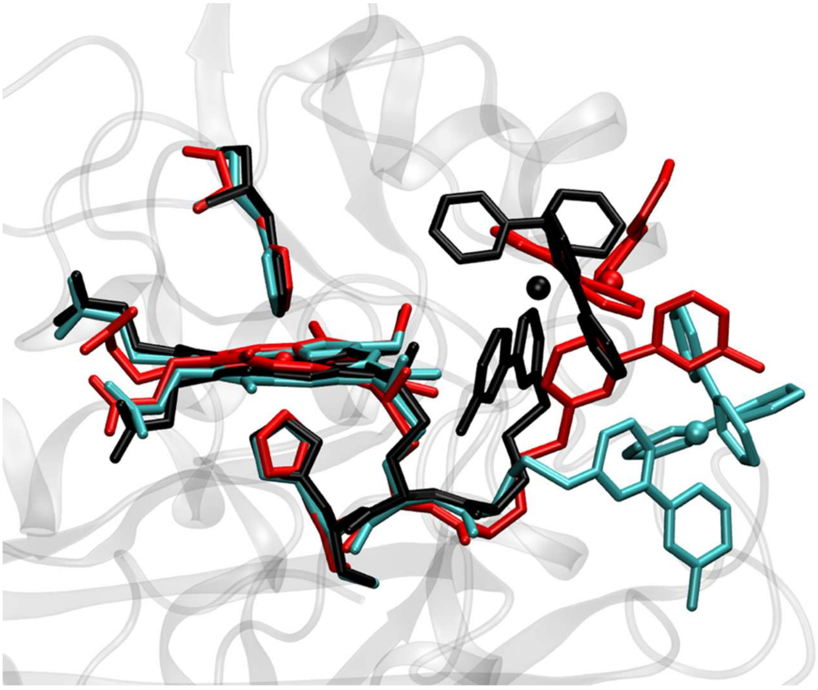

Fig. S13. Superposition of three of the eight conformers (conformers **1**, **2**, **5**) observed in molecular dynamics simulation of Ru-labeled MtrC. The three structures are aligned with respect to the Heme 10, shown in stick representation and color-coded by the conformation number: **1**, black; **2**, red; **5**, cyan. The protein backbone is depicted in grey. The edge-to-edge distances between Heme 10 and the Ru-label are 5.3, 5.6, 8.4 Angstroms for conformers **1**, **2**, **5**, respectively.

References

1. C.W.J. Lockwood, J.H. van Wonderen, M.J. Edwards, S.E.H. Piper, G.F. White, S. Newton-Payne, D.J. Richardson, T.A. Clarke, J.N. Butt, Membrane-spanning electron transfer proteins from electrogenic bacteria: production and investigation. *Methods Enzymol.* **613**, 257-275 (2018).
2. J.H. van Wonderen, D.B. Li, S.E.H. Piper, C.Y. Lau, L.P. Jenner, C.R. Hall, T.A. Clarke, N.J. Watmough, J.N. Butt, Photosensitised multiheme cytochromes as light-driven molecular wires and resistors. *ChemBioChem* **19**, 2206-2215 (2018).
3. I. Barr, F. Guo, Pyridine hemochromagen assay for determining the concentration of heme in purified protein solutions. *Bio Protoc.* **5**, e1594 (2015).
4. M.J. Edwards, G.F. White, M. Norman, A. Tome-Fernandez, E. Ainsworth, L. Shi, J.K. Fredrickson, J.M. Zachara, J.N. Butt, D.J. Richardson, T.A. Clarke, Redox linked flavin sites in extracellular decaheme proteins involved in microbe-mineral electron transfer. *Sci. Rep.* **5**, 11677 (2015).
5. P. Emsley, K. Cowtan, Coot: model-building tools for molecular graphics. *Acta Crystallogr. D* **60**, 2126-2132 (2004).
6. G.N. Murshudov, P. Skubak, A.A. Lebedev, N.S. Pannu, R.A. Steiner, R.A. Nicholls, M.D. Winn, F. Long, A.A. Vagin, REFMAC5 for the refinement of macromolecular crystal structures. *Acta Crystallogr. D* **67**, 355-367 (2011).
7. E. Krissinel, K. Henrick, Secondary-structure matching (SSM), a new tool for fast protein structure alignment in three dimensions. *Acta Crystallogr. D* **60**, 2256-2268 (2004).
8. B. Demeler (2005) UltraScan - A comprehensive data analysis software package for analytical ultracentrifugation experiments. *Analytical Ultracentrifugation: Techniques and Methods*, ed Scott DJ, Harding, S. E., and Rowe, A. J. (Royal Society of Chemistry, Cambridge, UK).
9. R.S. Hartshorne, B.N. Jepson, T.A. Clarke, S.J. Field, J. Fredrickson, J. Zachara, L. Shi, J.N. Butt, D.J. Richardson, Characterization of *Shewanella oneidensis* MtrC: a cell-surface decaheme cytochrome involved in respiratory electron transport to extracellular electron acceptors. *J. Biol. Inorg. Chem.* **12**, 1083-1094 (2007).

10. S.J. Marritt, G.L. Kemp, L. Xiaoe, J.R. Durrant, M.R. Cheesman, J.N. Butt, Spectroelectrochemical characterization of a pentaheme cytochrome in solution and as electrocatalytically active films on nanocrystalline metal-oxide electrodes. *J. Am. Chem. Soc.* **130**, 8588-8589 (2008).
11. L.P. Jenner, J.M. Kurth, S. van Helmont, K.P. Sokol, E. Reisner, C. Dahl, J.M. Bradley, J.N. Butt, M.R. Cheesman, Heme ligation and redox chemistry in two bacterial thiosulfate dehydrogenase (TsdA) enzymes. *J. Biol. Chem.* **294**, 18002-18014 (2019).
12. D. Stamou, D. Gourdon, M. Liley, N.A. Burnham, A. Kulik, H. Vogel, C. Duschl, Uniformly flat gold surfaces: imaging the domain structure of organic monolayers using scanning force microscopy. *Langmuir* **13**, 2425-2428 (1997).
13. V. Fourmond, K. Hoke, H.A. Heering, C. Baffert, F. Leroux, P. Bertrand, C. Leger, SOAS: A free program to analyze electrochemical data and other one-dimensional signals. *Bioelectrochem.* **76**, 141-147 (2009).
14. J.H. van Wonderen, C.R. Hall, X.Y. Jiang, K. Adamczyk, A. Carof, I. Heisler, S.E.H. Piper, T.A. Clarke, N.J. Watmough, I.V. Sazanovich, M. Towrie, S.R. Meech, J. Blumberger, J.N. Butt, Ultrafast light-driven electron transfer in a Ru(II)tris(bipyridine)-labeled multiheme cytochrome. *J. Am. Chem. Soc.* **141**, 15190-15200 (2019).
15. S.E. Bettis, D.M. Ryan, M.K. Gish, L. Alibabaei, T.J. Meyer, M.L. Waters, J.M. Papanikolas, Photophysical characterization of a helical peptide chromophore-water oxidation catalyst assembly on a semiconductor surface using ultrafast spectroscopy. *J. Phys. Chem. C* **118**, 6029-6037 (2014).
16. D.F. Zigler, Z.A. Morseth, L. Wang, D.L. Ashford, M.K. Brennaman, E.M. Grumstrup, E.C. Brigham, M.K. Gish, R.J. Dillon, L. Alibabaei, G.J. Meyer, T.J. Meyer, J.M. Papanikolas, Disentangling the physical processes responsible for the kinetic complexity in interfacial electron transfer of excited Ru(II) polypyridyl dyes on TiO_2. *J. Am. Chem. Soc.* **138**, 4426-4438 (2016).
17. L. Wang, D.L. Ashford, D.W. Thompson, T.J. Meyer, J.M. Papanikolas, Watching photoactivation in a Ru(II) chromophore-catalyst assembly on TiO_2 by ultrafast spectroscopy. *J. Phys. Chem. C* **117**, 24250-24258 (2013).
18. K. Kalyanasundaram, Photophysics, photochemistry and solar-energy conversion with tris(bipyridyl)Ruthenium(II) and its analogs. *Coord. Chem. Rev.* **46**, 159-244 (1982).
19. M.Z. Hoffman, M.G. Simic, Q.G. Mulazzani, S. Emmi, P.G. Fuochi, M. Venturi, One-Electron Reduction of 2,2'-Bipyridine in Aqueous-Solution. *Radiat. Phys. Chem.* **12**, 111-113 (1978).
20. S.A. McFarland, K.A.W.Y. Cheng, F.S. Lee, F.L. Cozens, N.P. Schepp, Nonthermalized excited states in Ru(II) polypyridyl complexes probed by ultrafast transient absorption spectroscopy with high photon energy excitation. *Can. J. Chem.* **86**, 1118-1125 (2008).
21. X.Y. Jiang, B. Burger, F. Gajdos, C. Bortolotti, Z. Futera, M. Breuer, J. Blumberger, Kinetics of trifurcated electron flow in the decaheme bacterial proteins MtrC and MtrF. *Proc. Natl. Acad. Sci. U.S.A.* **116**, 3425-3430 (2019).
22. Z. Futera, J. Blumberger, Electronic couplings for charge transfer across molecule/metal and molecule/semiconductor interfaces: performance of the projector operator-based diabatization approach. *J. Phys. Chem. C* **121**, 19677-19689 (2017).
23. J. Blumberger, Free energies for biological electron transfer from QM/MM calculation: method, application and critical assessment. *Phys. Chem. Chem. Phys.* **10**, 5651-5667 (2008).
24. M. Breuer, P. Zarzycki, L. Shi, T.A. Clarke, M.J. Edwards, J.N. Butt, D.J. Richardson, J.K. Fredrickson, J.M. Zachara, J. Blumberger, K.M. Rosso, Molecular structure and free energy landscape for electron transport in the decahaem cytochrome MtrF. *Biochem. Soc. Trans.* **40**, 1198-1203 (2012).
25. R. Dennington, T. Keith, M. J., GaussView Version 5. Semichem Inc. Shawnee Mission KS. (2009).
26. J.C. Phillips, R. Braun, W. Wang, J. Gumbart, E. Tajkhorshid, E. Villa, C. Chipot, R.D. Skeel, L. Kale, K. Schulten, Scalable molecular dynamics with NAMD. *J. Comput. Chem.* **26**, 1781-1802 (2005).
